# Adenosine and hyaluronan promote lung fibrosis and pulmonary hypertension in combined pulmonary fibrosis and emphysema

**DOI:** 10.1242/dmm.038711

**Published:** 2019-05-15

**Authors:** Scott D. Collum, Jose G. Molina, Ankit Hanmandlu, Weizhen Bi, Mesias Pedroza, Tinne C. J. Mertens, Nancy Wareing, Wang Wei, Cory Wilson, Wenchao Sun, Jayakumar Rajadas, Paul L. Bollyky, Kemly M. Philip, Dewei Ren, Rajarajan A. Thandavarayan, Brian A. Bruckner, Yang Xia, Michael R. Blackburn, Harry Karmouty-Quintana

**Affiliations:** 1 Department of Biochemistry and Molecular Biology, McGovern Medical School, University of Texas Health Science Center at Houston, Houston, TX 77030, USA; 2 Department of Medicine, Baylor College of Medicine, Houston, TX 77030, USA; 3 Biomaterials and Advanced Drug Delivery Lab, Stanford University School of Medicine, Stanford, CA 94304, USA; 4 Division of Infectious Diseases, Department of Medicine, Stanford University School of Medicine, Stanford, CA 94305, USA; 5 Houston Methodist DeBakey Transplant Center, Houston Methodist Hospital, Houston, TX 77030, USA

**Keywords:** Group III PH, Hyaluronan, Lung fibrosis, Pulmonary hypertension, Adenosine, Airspace enlargement, Macrophages, Hyaluronan synthases

## Abstract

Combined pulmonary fibrosis and emphysema (CPFE) is a syndrome that predominantly affects male smokers or ex-smokers and it has a mortality rate of 55% and a median survival of 5 years. Pulmonary hypertension (PH) is a frequently fatal complication of CPFE. Despite this dismal prognosis, no curative therapies exist for patients with CPFE outside of lung transplantation and no therapies are recommended to treat PH. This highlights the need to develop novel treatment approaches for CPFE. Studies from our group have demonstrated that both adenosine and its receptor ADORA2B are elevated in chronic lung diseases. Activation of ADORA2B leads to elevated levels of hyaluronan synthases (HAS) and increased hyaluronan, a glycosaminoglycan that contributes to chronic lung injury. We hypothesize that ADORA2B and hyaluronan contribute to CPFE. Using isolated CPFE lung tissue, we characterized expression levels of ADORA2B and HAS. Next, using a unique mouse model of experimental lung injury that replicates features of CPFE, namely airspace enlargement, PH and fibrotic deposition, we investigated whether 4MU, a HAS inhibitor, was able to inhibit features of CPFE. Increased protein levels of ADORA2B and HAS3 were detected in CPFE and in our experimental model of CPFE. Treatment with 4MU was able to attenuate PH and fibrosis but not airspace enlargement. This was accompanied by a reduction of HAS3-positive macrophages. We have generated pre-clinical data demonstrating the capacity of 4MU, an FDA-approved drug, to attenuate features of CPFE in an experimental model of chronic lung injury.

This article has an associated First Person interview with the first author of the paper.

## Abstract (precis)

**Summary:** Fibrotic deposition and PH are inhibited by the FDA-approved drug hymecromone, suggesting hyaluronan synthesis inhibition as a potential therapy for CPFE and highlighting a novel mechanism through HAS3-positive macrophages.

## INTRODUCTION

Chronic obstructive pulmonary disease (COPD) and idiopathic pulmonary fibrosis (IPF) embody the third leading cause of death in the US (https://www.cdc.gov/nchs/data/hus/2017/019.pdf). COPD and IPF represent two distinct multifactorial chronic lung diseases that are characterized by distinct clinical and pathological hallmarks. A defining pathological hallmark of COPD is the presence of emphysema, defined as airspace enlargement as a result of destruction of the alveoli (Hogg and Timens, 2009; Pauwels et al., 2001). IPF is characterized by varying degrees of inflammation, aberrant fibroblast proliferation and extracellular matrix deposition that result in distortion of pulmonary architecture and loss of pulmonary function (Lynch and Toews, 1998; Panos et al., 1990; Sime and O'Reilly, 2001; Thannickal et al., 2004).

Traditionally, COPD (specifically emphysema) and IPF are regarded as separate disease entities. Nonetheless, hallmarks of both COPD and IPF were first described in autopsies of lungs in 1974 (Auerbach et al., 1974); however, it was not until 2005 that this condition was classified as a well-defined syndrome termed ‘combined pulmonary fibrosis and emphysema’ (CPFE) (Cottin et al., 2005). Male smokers or ex-smokers most often present with CPFE (Cottin et al., 2005; Jankowich and Rounds, 2010, 2012) and it has a dismal prognosis, with only a 55% survival rate at 5 years (Cottin et al., 2005). CPFE is a distinct under-recognized syndrome for which no specific diagnostic guidelines are present, yet studies show that its presentation is much more frequent than was previously believed (Cottin, 2013). An important and frequently fatal co-morbidity in CPFE is the development of pulmonary hypertension (PH), which is defined by a mean pulmonary arterial pressure (mPAP) of ≥25 mmHg at rest (Archer et al., 2010). The prevalence of PH in CPFE ranges between 47-90% and is much higher than in patients with IPF or emphysema alone (Caminati et al., 2013; Cottin, 2013; Cottin et al., 2010; Seeger et al., 2013). Confirmation of PH by right-heart catheterization data reduces 1-year survival rates to only 60% (Cottin et al., 2010). Despite this dismal prognosis, no therapy options exist for patients with CPFE outside of smoking cessation, oxygen therapy and lung transplantation (Jankowich and Rounds, 2012; Lin and Jiang, 2015). Specifically for PH in CPFE, endothelin receptor antagonists, prostanoids and phosphodiesterase type 5 inhibitors have been used. However, these agents are not recommended for PH in CPFE because of their vasodilation profile that results in ventilation/perfusion mismatch (Seeger et al., 2013), highlighting the need to develop novel treatment approaches for CPFE.

Although several gene mutations have been associated with CPFE, including telomerase genes [TERT or TR (also known as TERC)], surfactant protein C (SFTPC) and ATP binding cassette subfamily A member 3 (ABCA3), these have been performed in very small familial cohorts (TERT/TR) or in case studies (SFTPC/ABCA3) (Lin and Jiang, 2015). As such, no single gene defect is known be associated with CPFE, and the mechanisms leading to the development of CPFE remain vastly unknown (Jankowich and Rounds, 2012). Despite this, studies from our group in COPD and IPF patients with PH have revealed increased activation of the adenosinergic axis and enhanced levels of the glycosaminoglycan hyaluronan (Garcia-Morales et al., 2016; Karmouty-Quintana et al., 2013a, 2012). In these studies, increased activation of the adenosine A2B receptor (ADORA2B) was shown to enhance expression of the hyaluronan synthases (HAS), contributing to the pathophysiology of chronic lung disease (Garcia-Morales et al., 2016; Karmouty-Quintana et al., 2015, 2013a, 2012). Furthermore, several studies from our group have implicated macrophages in the pathophysiology of lung fibrosis and PH. In these studies, deletion of myeloid ADORA2B was able to prevent BLM-induced PH and lung fibrosis (Karmouty-Quintana et al., 2015). Further studies from our group have also demonstrated an important link between macrophage activation and ADORA2B expression promoting fibrotic lung injury (Philip et al., 2017). These results point at macrophages as a potential mechanism promoting lung fibrosis and PH; however, whether they play a role in CPFE is not known.

Using a unique model of experimental lung injury that replicates features of CPFE, namely airspace enlargement, PH and fibrotic deposition (Chunn et al., 2005; Karmouty-Quintana et al., 2013a), we investigated the capacity of an HAS inhibitor to attenuate features of chronic lung injury and determined a link between hyaluronan expression and macrophages.

## RESULTS

### Characterization of our experimental model of CPFE

We used *Ada^−/−^* mice to model features of CPFE. *Ada^−/−^* mice received supplemental PEG-ADA, allowing them to live normally, from birth up to week 24. Starting on week 24, PEG-ADA was gradually reduced over 9 weeks and, starting on week 34, mice were provided with either control chow or were medicated with 4MU for 4 weeks. The gradual reduction of PEG-ADA results in accumulation of extracellular adenosine that is associated with chronic injury (Karmouty-Quintana et al., 2013b).

**Fig. 1.**
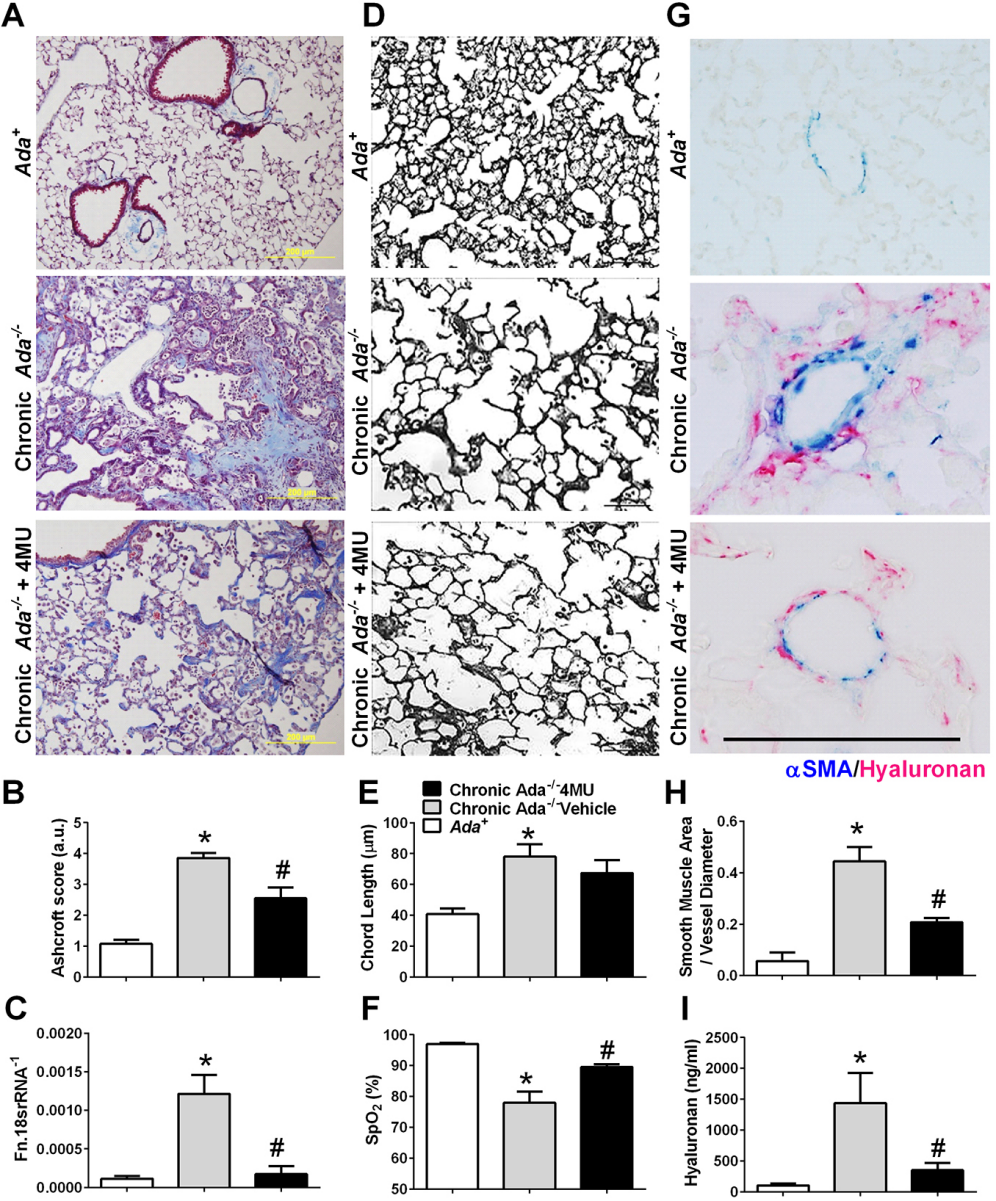
**Features of chronic lung injury in *Ada^−/−^* mice and the effect of 4MU or control chow.** (A) Representative Masson's Trichrome images showing fibrotic deposition in *Ada^−/−^* mice (middle panel) compared to *Ada^+^* mice (upper panel). The effect of 4MU in *Ada^−/−^* mice is shown in the bottom panel. (B,C) Ashcroft scores from Masson's Trichrome images (B) and mRNA expression levels of fibronectin (C) from *Ada^+^* (white bars), *Ada^−/−^* (gray bars) and *Ada^−/−^*+4MU-treated (black bars) mice. (D) Black and white images of the lung parenchyma from *Ada^+^* mice (upper panel), *Ada^−/−^* (middle panel) and *Ada^−/−^*+4MU-treated (lower panel) mice. (E,F) Mean chord length determined histologically (E) and arterial oxygen saturation (F) from *Ada^+^* (white bars), *Ada^−/−^* (gray bars) and *Ada^−/−^*+4MU-treated mice (black bars). (G) Representative dual-IHC staining for αSMA (blue) and hyaluronan (magenta) showcasing arterioles from *Ada^+^* (upper panel), *Ada^−/−^* (middle panel) and *Ada^−/−^*+4MU-treated (lower panel) mice. (H,I) Morphometric evaluation of αSMA deposition from five to seven vessels for each mouse (H) and BALF levels of hyaluronan (I) from *Ada^+^* (white bars), *Ada^−/−^* (gray bars) and *Ada^−/−^*+4MU-treated (black bars) mice. Data are mean+s.e.m. (*n*=5 per group). *P<0.05 (comparisons between *Ada^+^* and *Ada^−/−^* treatment groups), ^#^*P*<0.05 (comparisons between *Ada^−/−^* and *Ada^−/−^*+4MU treatment groups); two-way ANOVA with the Benjamin, Krieger and Yekutieli post hoc test. Scale bars: 200 µm.

A hallmark of CPFE is the presence of fibrotic deposition and airspace enlargement. We determined the extent of fibrotic deposition first by staining lung sections with Masson's Trichrome and performing Ashcroft scores to determine the extent of fibrosis. These experiments revealed a marked increase in fibrotic deposition in *Ada^−/−^* mice compared to *Ada^+^* mice, which was significantly attenuated in *Ada-*deficient mice treated with the hyaluronan inhibitor 4-methylumbelliferone (4MU; Fig. 1A,B). These histological changes were consistent with expression levels of fibronectin that were elevated in *Ada^−/−^* mice compared to *Ada*-competent mice. Therapy with 4MU reduced *Fn1* expression levels in *Ada^−/−^* mice (Fig. 1C). We next examined the extent of airspace enlargement, a key feature of CPFE, in our mouse model using black and white images of the lung parenchyma. *Ada^−/−^* mice presented with evidence of airspace enlargement, as observed histologically, and by mean chord length measurements, determined morphometrically, in comparison to *Ada^+^* mice (Fig. 1D,E). Treatment with 4MU did not alter the emphysematous development in *Ada*-deficient mice (Fig. 1D,E), despite improved arterial oxygenation (SpO_2_) compared to *Ada^−/−^* mice exposed to control chow (Fig. 1F). The development of PH is a serious and common complication of CPFE (Cottin et al., 2010). A feature of PH associated with chronic lung disease is vascular remodeling and hyaluronan deposition (Collum et al., 2017; Karmouty-Quintana et al., 2013a, 2012). In order to assess the extent of vascular remodeling, we performed dual-immunohistochemistry (IHC) for alpha smooth muscle actin (αSMA; Acta2) and hyaluronan. These experiments revealed extensive muscularization of arterioles in *Ada^−/−^* compared to *Ada^+^* mice that was significantly attenuated in 4MU-treated *Ada^−/−^* mice (Fig. 1G). Widespread hyaluronan deposition was observed surrounding remodeled vessels in *Ada^−/−^* mice, whereas no hyaluronan was present in parenchymal vessels of *Ada*-competent animals; 4MU treatment drastically reduced the levels of peri-vascular hyaluronan in mice lacking *Ada* (Fig. 1G). These observations were backed by morphometric evaluation of vascular wall remodeling and hyaluronan levels in bronchoalveolar lavage fluid (BALF) (Fig. 1H,I). These analyses demonstrated increased αSMA signals in the remodeled vessels of *Ada^−/−^* mice compared to *Ada^+^* mice that were attenuated in *Ada^−/−^* mice exposed to 4MU (Fig. 1H). BALF hyaluronan levels revealed increased hyaluronan levels in *Ada^−/−^* compared to control mice that were markedly reduced in 4MU-treated *Ada^−/−^* mice (Fig. 1I). Taken together, our results show that our model of *Ada^−/−^* mice presents with cardinal features of CPFE: fibrotic deposition, airspace enlargement and vascular remodeling, a key component of PH. Furthermore, we demonstrate that treatment of these mice with 4MU is able to attenuate both the fibrotic deposition and vascular remodeling in *Ada^−/−^* mice. To ensure that mice ingested 4MU, we measured levels of its main metabolite, 4-methylumbelliferyl-β-D-glucuronide hydrate (4MUG), in plasma from 4MU-treated mice. These results demonstrate increased 4MUG levels in mice treated with 4MU (Fig. S1).

### Vascular physiology, metabolism and hyaluronan levels in *Ada*^−/−^ mice

**Fig. 2.**
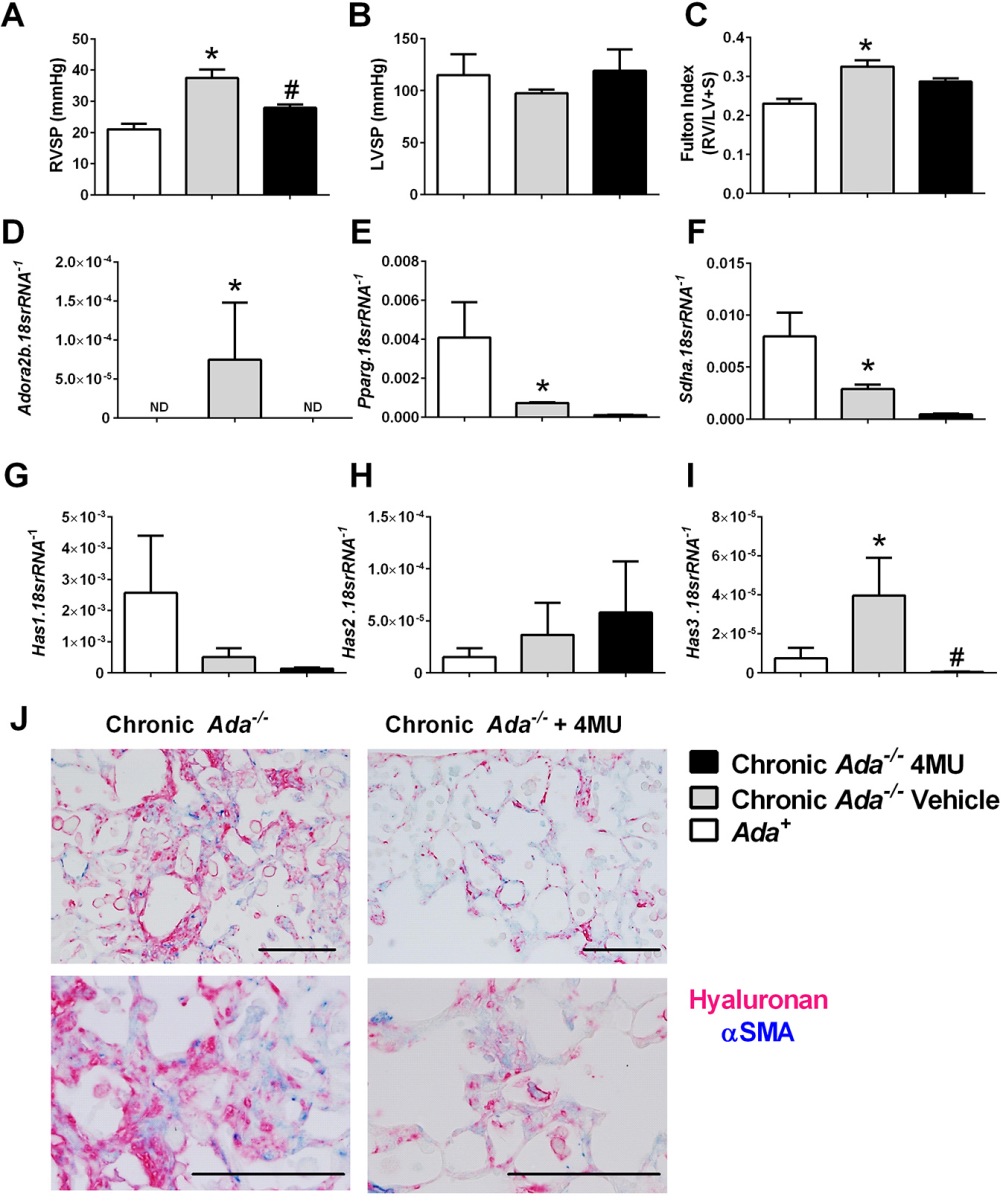
**Cardiovascular physiology, metabolic profile, and hyaluronan synthesis and deposition in *Ada^−/−^*.** (A-C) Right ventricular systolic pressure (RVSP; A), left ventricular systolic pressure (LVSP; B) and Fulton index ratio (C) determined by the measurement of right ventricle and left ventricle with septum from *Ada^+^* (white bars), *Ada^−/−^* (gray bars) and *Ada^−/−^*+4MU-treated (black bars) mice. (D-I) Transcript expression levels from lung tissue for *Adora2b* (D), *Pparg* (E), *Sdha* (F), *Has1* (G), *Has2* (H), *Has3* (I) by RT-PCR from *Ada^+^* (white bars), *Ada^−/−^* (gray bars) and *Ada^−/−^*+4MU-treated (black bars) mice. (J) Representative dual-IHC staining in fibrotic areas of *Ada^−/−^* or *Ada^−/−^*+4MU treatment groups for αSMA (blue) and hyaluronan (magenta). Data are mean+s.e.m. (*n*=5 per group). *P<0.05 (comparisons between *Ada^+^* and *Ada^−/−^* treatment groups), ^#^*P*<0.05 (comparisons between *Ada^−/−^* and *Ada^−/−^*+4MU treatment groups); two-way ANOVA with the Benjamin, Krieger and Yekutieli post hoc test. Scale bars: 200 µm.

Although vascular remodeling is a key feature of PH, right-heart catheterization and determination of right ventricle systolic pressure (RVSP) in mice is necessary to adequately evaluate the extent of PH. As such, RVSP measurements were determined in our experimental model. We report increased RVSP in *Ada^−/−^* mice compared to *Ada^+^* mice, which was attenuated in 4MU-treated *Ada^−/−^* mice (Fig. 2A). Consistent with the phenomenon of PH, no differences in left ventricle systolic pressure (LVSP) were observed between treatment groups (Fig. 2B). Our RVSP data was accompanied by evidence of right ventricle hypertrophy (RVH) in *Ada^−/−^* mice compared to *Ada^+^* littermates that did not appear to be significantly attenuated following 4MU therapy (Fig. 2C). In order to probe the mechanisms associated with the development of PH, we next evaluated the expression levels of the adenosine A2B receptor (*Adora2b*) and evidence of metabolic alterations. Enhanced adenosinergic axis and metabolic alterations have been reported in Group III PH (Garcia-Morales et al., 2016; Karmouty-Quintana et al., 2015, 2013a, 2012). These experiments revealed heightened *Adora2b* expression in *Ada^−/−^* mice but no expression in *Ada^+^* or 4MU-treated *Ada^−/−^* mice (Fig. 2D). Consistent with dysregulated metabolism, we report reduced levels of *Pparg* and *Sdha* that were not rescued following 4MU therapy (Fig. 2E,F). We next evaluated the expression levels of HAS, which are known to be upregulated by ADORA2B activation. These experiments revealed elevated expression of *Has3*, but not *Has1* or *Has2*, in *Ada^−/−^* mice compared to controls that was significantly reduced following 4MU treatment in Ada-deficient mice (Fig. 2G-I). Dual-IHC for hyaluronan and αSMA revealed increased hyaluronan deposition in fibrotic areas in *Ada^−/−^* mice that was significantly attenuated following 4MU therapy (Fig. 2J). These results demonstrate that *Ada^−/−^* mice present with physiological features of PH including elevated RVSP and RVH in addition to increased *Adora2b* and *Has3* expression, concomitant with elevated hyaluronic acid deposition observed histologically. 4MU therapy was able to attenuate the increased RVSP and hyaluronic acid deposition associated with reduced *Adora2b* and *Has3* expression levels, but did not affect the dysregulated metabolic profile, nor reverse RVH.

### Macrophages in *Ada*^−/−^ mice

**Fig. 3.**
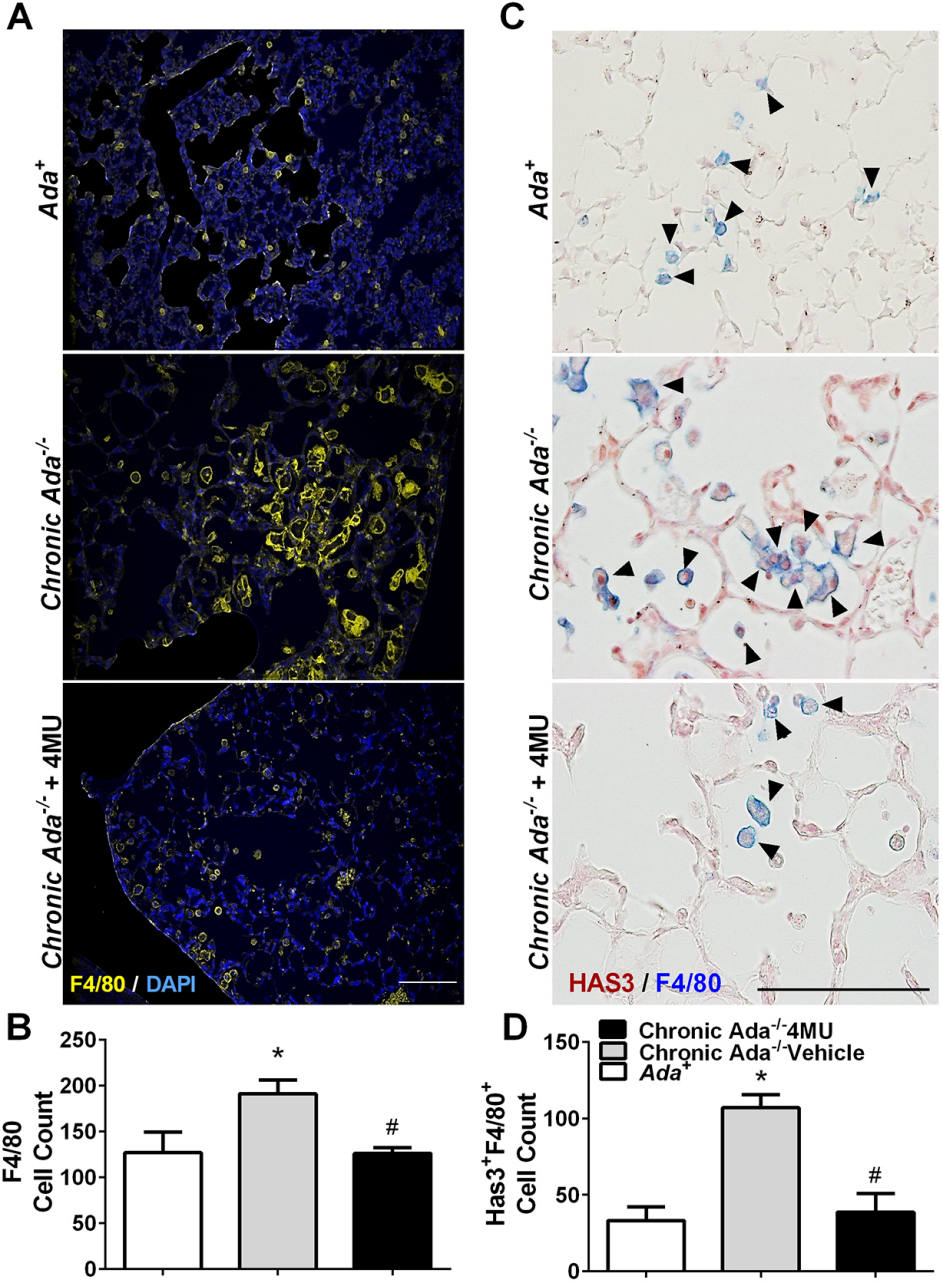
**Macrophages in *Ada^−/−^* mice.** (A) IHC for F4/80 (yellow) and nuclear DAPI stain showing F4/80-positive cells in *Ada^+^* (upper panel), *Ada^−/−^* (middle panel) and *Ada^−/−^*+4MU-treated (lower panel) mice. (B) F4/80-positive cells from ten micropictographs from the lung parenchyma from *Ada^+^* (white bars), *Ada^−/−^* (gray bars) and *Ada^−/−^*+4MU-treated (black bars) mice. (C) Dual-IHC for HAS3 (red/brown) and F4/80 (blue) in *Ada^+^* (upper panel), *Ada^−/−^* (middle panel) and *Ada^−/−^*+4MU (lower panel). Arrowheads indicate F4/80-positive cells. (D) Double F4/80- and HAS3-positive cells from ten micropictographs from the lung parenchyma from *Ada^+^* (white bars), *Ada^−/−^* (gray bars) and *Ada^−/−^*+4MU-treated (black bars) mice. Data are mean+s.e.m. (*n*=5 per group). *P<0.05 (comparisons between *Ada^+^* and *Ada^−/−^* treatment groups), ^#^*P*<0.05 (comparisons between *Ada^−/−^* and *Ada^−/−^*+4MU treatment groups); two-way ANOVA with the Benjamin, Krieger and Yekutieli post hoc test. Scale bars: 75 µm in A; 200 µm in C.

**Fig. 4.**
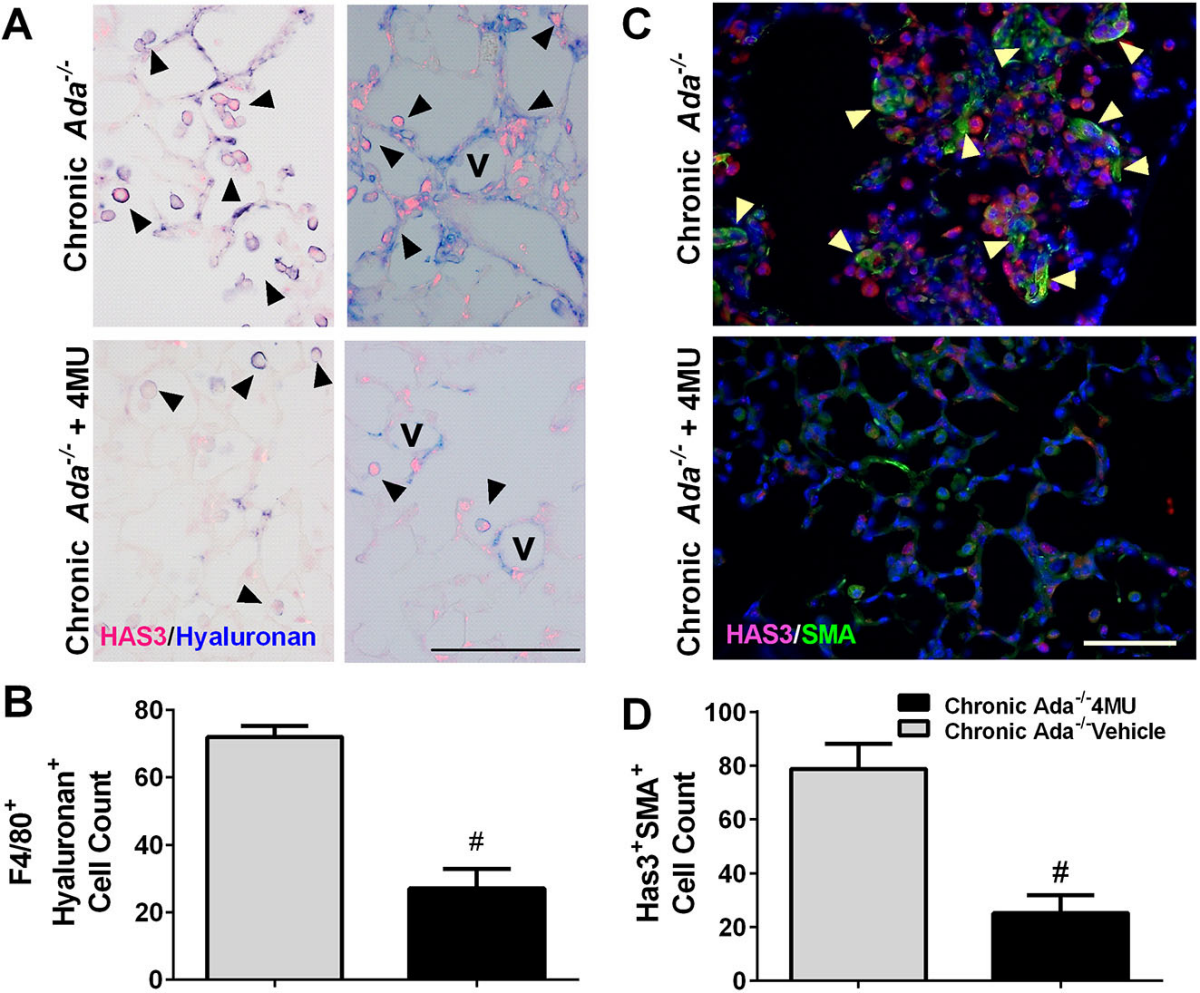
**Macrophages in *Ada^−/−^* mice express HAS3.** (A) Dual-IHC for HAS3 (magenta) and hyaluronan (blue) in *Ada^−/−^* (upper panels) and *Ada^−/−^*+4MU-treated (lower panels) mice. Black arrowheads indicate αSMA-positive signals. V, vessel. (B) Double HAS3- and hyaluronan-positive cells from ten micropictographs from the lung parenchyma from *Ada^−/−^* (gray bar) and *Ada^−/−^*+4MU-treated (black bar) mice. (C) Dual-IHC for HAS3 (magenta) and αSMA (green) in *Ada^−/−^* (upper panels) and *Ada^−/−^*+4MU-treated (lower panels) mice. Yellow arrowheads indicate αSMA-positive signals. (D) Double HAS3- and αSMA-positive cells from ten micropictographs from the lung parenchyma from *Ada^−/−^* (gray bar) and *Ada^−/−^*+4MU-treated (black bar) mice. Data are mean+s.e.m. (*n*=5 per group). ^#^*P*<0.05 (comparisons between *Ada^−/−^* and *Ada^−/−^*+4MU treatment groups); unpaired two-tailed Student's *t*-test with a Welch correction. Scale bar: 100 µm in A; 75 µm in C.

**Fig. 5.**
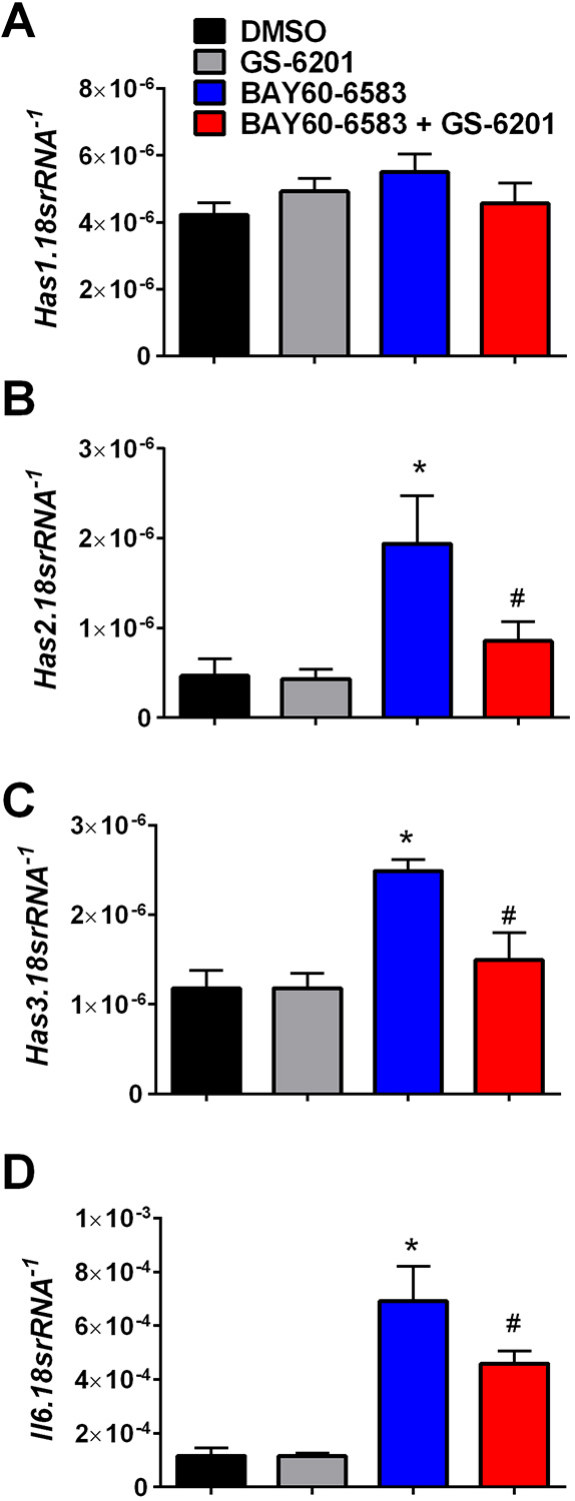
**Response of macrophages to an ADORA2B agonist.** (A-D) Transcript levels of *Has1* (A), *Has2* (B), *Has3* (C) and *IL6* (D) of MH-S cells exposed to DMSO (black bars), the ADORA2B antagonist GS-6201 (gray bars), the ADORA2B agonist BAY60-6583 (blue bars) or under the presence of both GS-6201 and BAY60-6583 (red bars). *P<0.05 (comparisons between DMSO and BAY60-6583 treatment groups). ^#^*P*<0.05 (comparisons between BAY60-6583 and BAY60-6583+GS-6201 treatment groups); two-way ANOVA with the Benjamin, Krieger and Yekutieli post hoc test. Data are mean+s.e.m. (*n*=5 for all groups).

An interesting feature of the *Ada^−/−^* mice was the presence of F4/80-positive cells in the lung (Fig. 3A), whereas treatment with 4MU resulted in an attenuation of F4/80-positive cells in *Ada^−/−^* mice (Fig. 3A,B). We next evaluated whether these cells were positive for HAS3. Indeed, dual-IHC for HAS3 and F4/80 revealed signals for HAS3 and F4/80 in *Ada^−/−^* mice that appeared to be reduced following treatment with 4MU (Fig. 3C,D). Interestingly, increased HAS3 signals were observed in vascular and epithelial cells. Further IHC demonstrated that these HAS3-positive cells also expressed hyaluronan and were observed close to vessels in *Ada^−/−^* mice (Fig. 4A). Treatment with 4MU resulted in an attenuation of HAS3/hyaluronan-positive cells and a marked reduction of hyaluronan (Fig. 4A,B). These HAS3-positive cells were also observed in areas rich in myofibroblasts (observed using αSMA immunofluorescence) that were reduced following 4MU treatment (Fig. 4C,D). Our group has previously demonstrated that activation of hypoxic adenosinergic axis in macrophages is important for the development of lung fibrosis (Karmouty-Quintana et al., 2015; Philip et al., 2017). In these studies, reduced hyaluronan deposition was observed in mice that lacked *Adora2b* in myeloid cells (Karmouty-Quintana et al., 2015). Thus, using MH-S cells, a murine macrophage cell line, we next examined whether increased expression of the HAS isozymes was ADORA2B-dependent. These experiments revealed that *Has2* and *Has3*, but not *Has1*, were increased following treatment with the ADORA2B agonist Bay60-6583, and that this response was inhibited by the presence of the ADORA2B antagonist GS-6201 (Fig. 5A-C). This response was consistent with elevated BAY60-6583-induced interleukin 6 that was inhibited by GS-6201 (Fig. 5D). IL6 is a known pro-fibrotic mediator downstream of macrophage ADORA2B stimulation (Karmouty-Quintana et al., 2015; Le et al., 2014; Philip et al., 2017). Taken together, these results point at an ADORA2B-mediated increase of HAS3-expressing macrophages (F4/80-positive cells) that lead to increased hyaluronan deposition.

### The adenosinergic response in CPFE

**Fig. 6.**
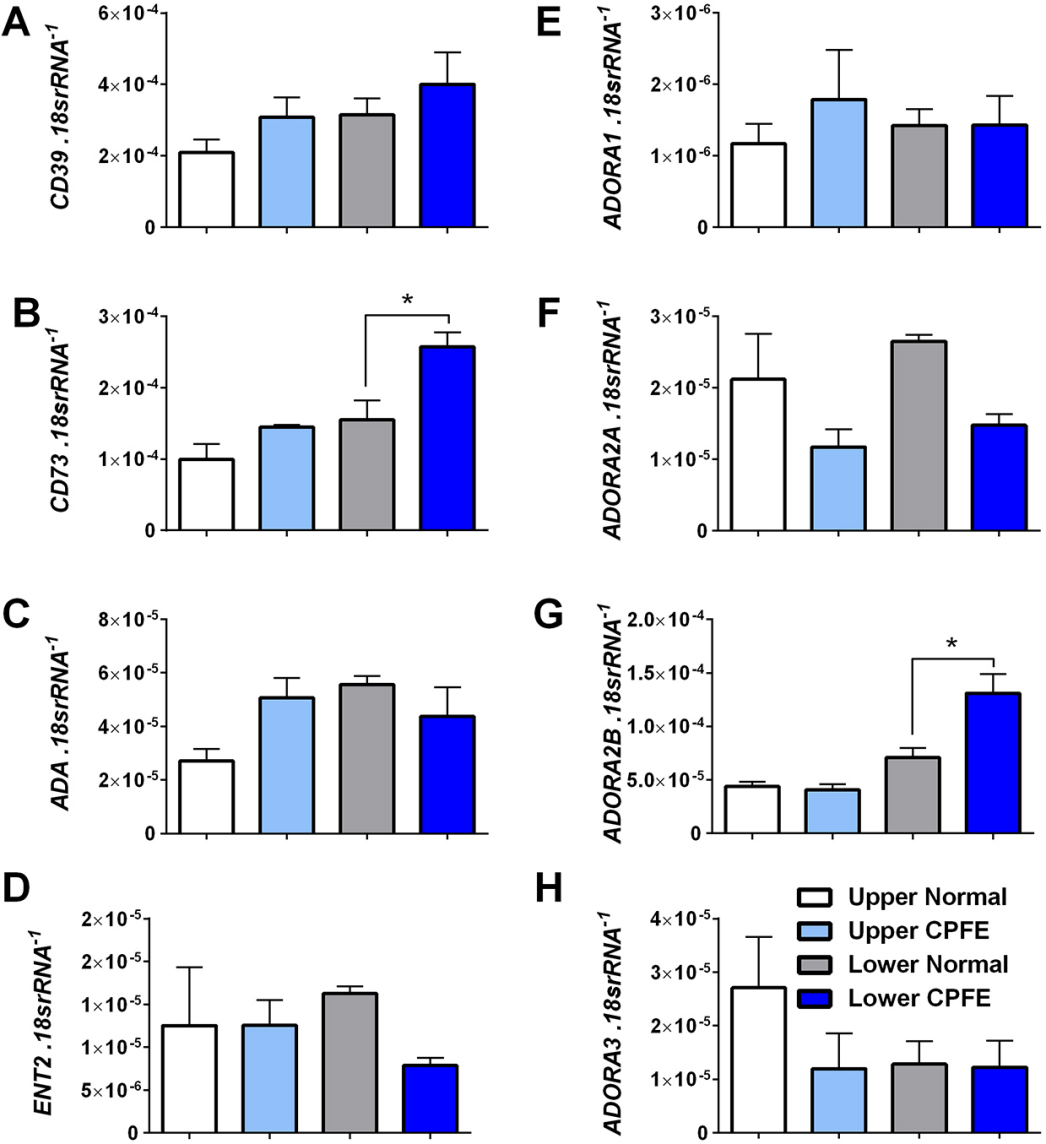
**The adenosinergic response in CPFE.** (A-H) Lung transcript levels for *CD39* (A), *CD73* (B), *ADA* (C), *ENT2* (D), *ADORA1* (E), *ADORA2A* (F), *ADORA2B* (G) and *ADORA3* (H) from upper lobes from normal lungs (white bars), upper lobes from CPFE lungs (light blue bars), lower lobes from normal lungs (gray bars) and lower lobes from CPFE lungs (dark blue bars). Data are mean+s.e.m. (*n*=3 per group). *P<0.05 (comparisons between normal and CPFE groups within upper or lower lung lobes); unpaired two-tailed Student's *t*-test with a Welch correction.

Using explanted lung tissue derived from upper or lower lung lobes from CPFE patients or from normal lungs that were discarded for transplantation, we evaluated mediators that are involved in the generation and degradation of adenosine, in addition to adenosine receptors. In CPFE, upper lobes often present with emphysema, whereas fibrotic deposition is more prevalent in lower lobes. Quantitative RT-PCR for the ectonucleotidases CD39 (ENTPD1) and CD73 (NT5E) (Fig. 6A,B) demonstrated increased expression of the rate limiting enzyme CD73 but not CD39 in lower lobes, but not in upper lobes, of CPFE compared to normal lung tissue (Fig. 6A,B). No differences were seen in *ADA* expression levels from lung tissue derived from upper or lower lobes (Fig. 6C). Equilibrative nucleoside transporters (ENT) also regulate extracellular adenosine levels whereby reduced expression is associated with increased adenosine accumulation and the development of lung injury (Luo et al., 2016). However, we report no difference in ENT expression in CPFE despite a downward trend for *ENT2* (*SLC29A2*) in lower lobes of CPFE lungs (Fig. 6D). Expression levels of adenosine receptors did not show significant alterations in *ADORA1* or *ADORA2A* between normal and CPFE lung tissue (Fig. 6E,F). However, increased expression of *ADORA2B* was detected in lower lobes of CPFE compared to normal lungs, but not in tissue derived from upper lobes (Fig. 6G). Similarly to *ADORA1* and *ADORA2A*, no significant difference in expression of *ADORA3* was observed between normal and CPFE-derived lung tissue (Fig. 6H). Taken together, these results suggest that in CPFE, as in other chronic lung diseases (Karmouty-Quintana et al., 2013b), adenosine synthesis is increased, together with expression of its low-affinity receptor, ADORA2B.

### The extracellular matrix in CPFE

**Fig. 7.**
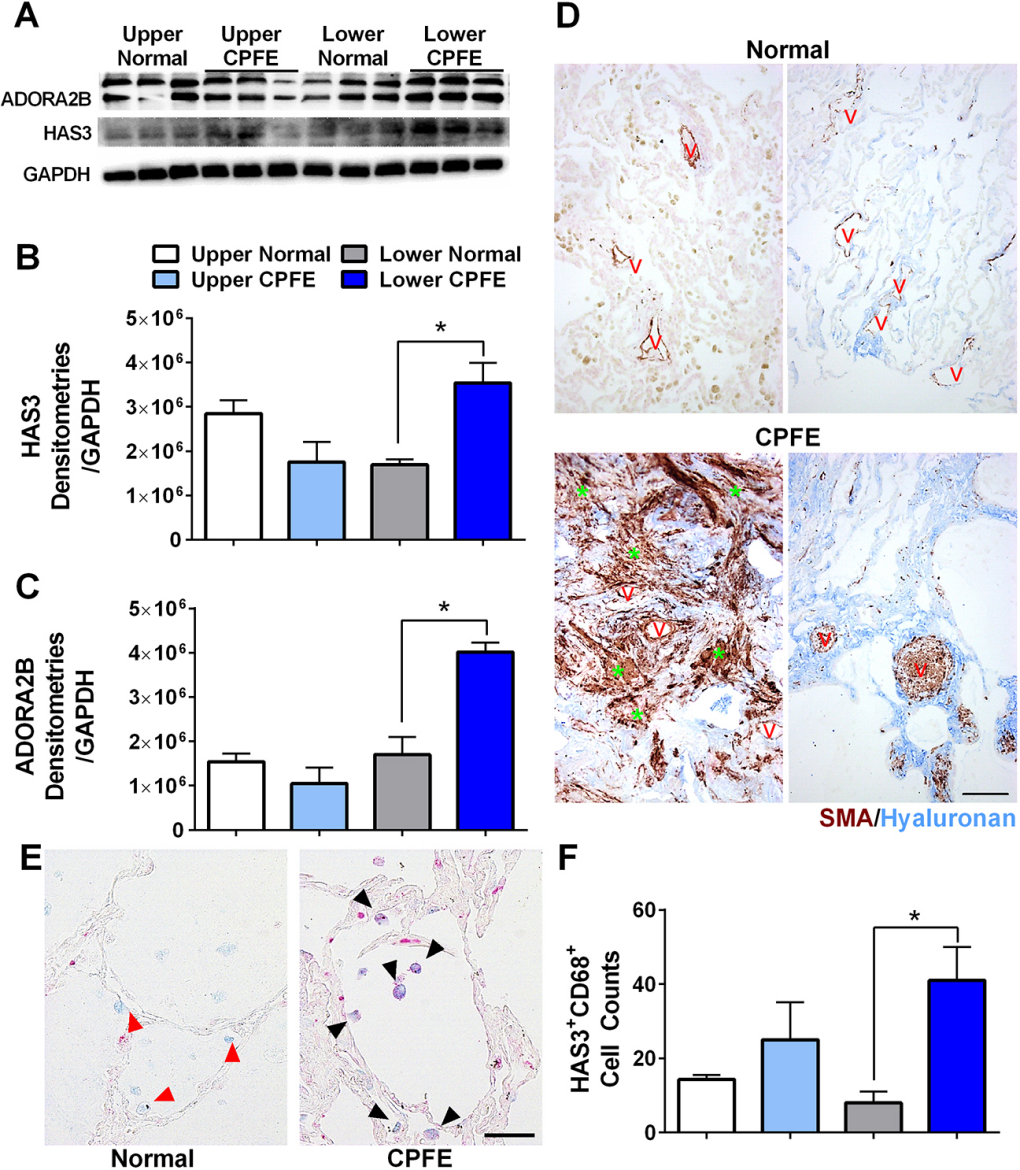
**ADORA2B and HAS3 are elevated in CPFE.** (A) Western blot for ADORA2B, HAS3 and GAPDH from upper and lower lobes from normal or CPFE lungs. (B,C) Densitometries for HAS3 (B) and ADORA2B (C) from upper lobes from normal lungs (white bars), upper lobes from CPFE lungs (light blue bars), lower lobes from normal lungs (gray bars) and lower lobes from CPFE lungs (dark blue bars). (D) Dual-IHC for αSMA (red/brown) and hyaluronan (blue) in normal (upper panels) or CPFE (lower panels) lungs. The red V denotes vessels and the green asterisks indicate myofibroblasts. (E) IHC for CD68 (blue) and HAS3 (red) from lower lobes from normal (left) and CPFE (right) lungs. Red arrowheads represent CD68-positive (HAS3-negative) signals. Black arrowheads indicate dual HAS3 and CD68 signals. (F) Double HAS3- and CD68-positive cells from ten micropictographs from the upper lobes from normal lungs (white bars), upper lobes from CPFE lungs (light blue bars), lower lobes from normal lungs (gray bars) and lower lobes from CPFE lungs (dark blue bars). Data are mean+s.e.m. (*n*=3 per group). *P<0.05 (comparisons between normal and CPFE groups within upper or lower lung lobes); unpaired two-tailed Student's *t*-test with a Welch correction. Scale bars: 50 µm.

**Fig. 8.**
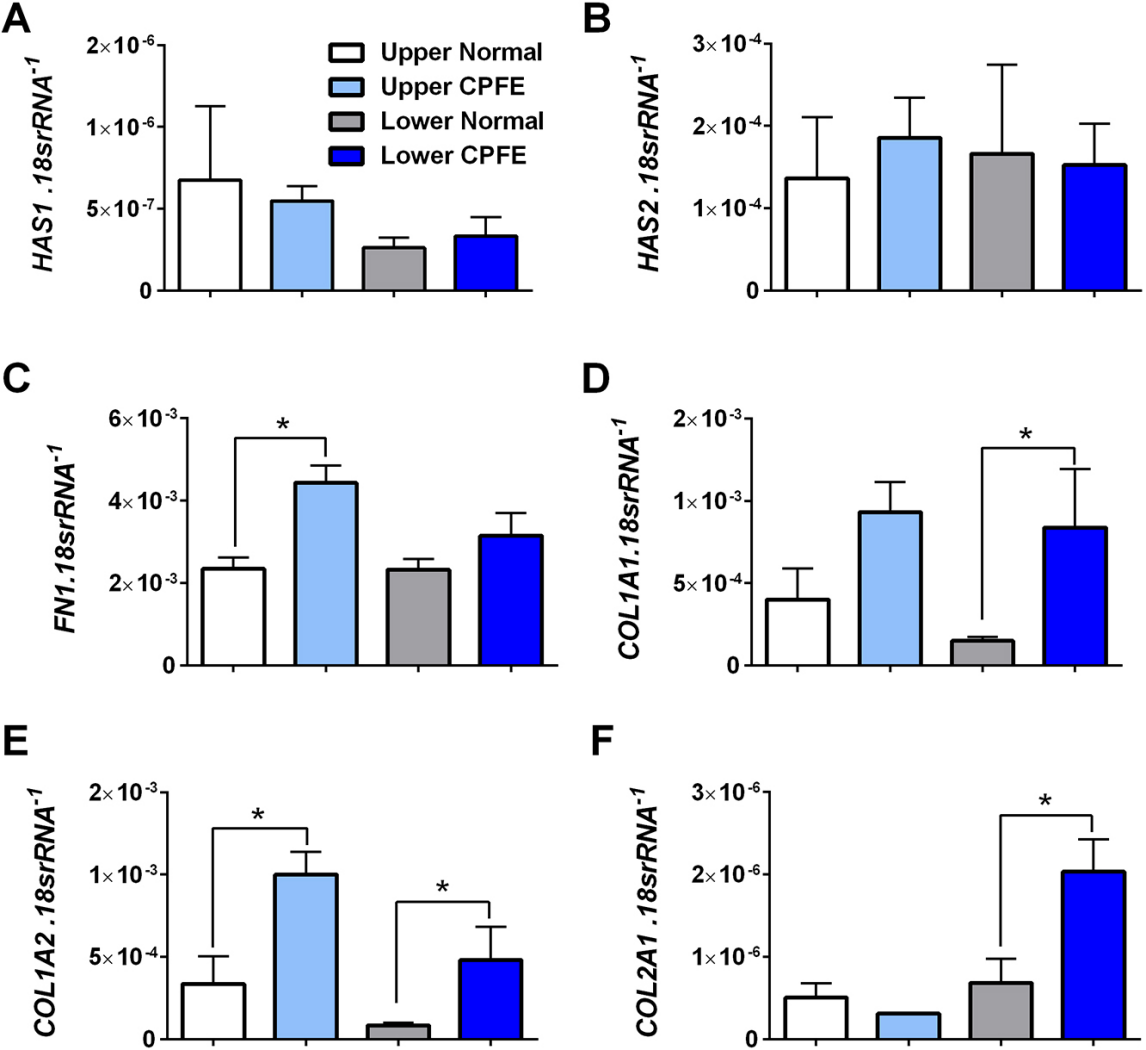
**Extracellular matrix signals in CPFE.** (A-F) Transcript levels for *HAS1* (A), *HAS2* (B), *FN1* (C), *COL1A1* (D), *COL1A2* (E) and *COL2A1* (F) from upper lobes from normal lungs (white bars), upper lobes from CPFE lungs (light blue bars), lower lobes from normal lungs (gray bars) and lower lobes from CPFE lungs (dark blue bars). Data are mean+s.e.m. (*n*=3 per group). *P<0.05 (comparisons between normal and CPFE groups within upper or lower lung lobes); unpaired two-tailed Student's *t*-test with a Welch correction.

Next, we evaluated the protein levels of ADORA2B and HAS3 in CPFE. Protein expression levels for ADORA2B revealed increased expression in lower lobes from CPFE lung tissue versus normal controls, concomitant with increased signals for HAS3 (Fig. 7A-C). These observations were in line with IHC for αSMA, revealing hyaluronan signals in highly remodeled areas of lung that are characterized by dense myofibroblast presence and prominent vascular remodeling in CPFE lungs compared to normal tissue (Fig. 7D). Similarly, staining for HAS3 and CD68 (a human monocytic phagocyte marker) revealed positive signals for CD68 and HAS3 in lower lobes of CPFE lungs (Fig. 7E,F). Our results did not show alterations in *HAS1* or *HAS2* between normal and CPFE lung tissue derived from upper or lower lobes (Fig. 8A,B). Gene expression for fibrotic genes revealed increased expression of *FN1* in upper lobes of CPFE versus normal lungs but not in lower lobes (Fig. 8C). We saw significantly increased expression of *COL1A1* in lung tissue derived from lower lobes from CPFE versus normal lung tissue (Fig. 8D). *COL1A2* was significantly elevated in both upper and lower lobes in CPFE versus normal lung tissue, whereas *COL2A1* was elevated only in the lower lobes from CPFE lungs (Fig. 8E,F). Interestingly, these results demonstrate that purinergic remodeling and increased ADORA2B levels in CPFE are observed in the lower lobes, in which fibrotic deposition is most evident, and in areas of vascular remodeling, in line with the role of adenosine in promoting fibrotic deposition and vascular remodeling in chronic lung injury (Karmouty-Quintana et al., 2013b).

## DISCUSSION

Despite reductions in mortality in cancer and cardiovascular disease, mortality rates for chronic lung diseases, including interstitial lung disease, have remained unaffected over the last decade (The Lancet Respiratory Medicine, 2016). This is a direct result of the disparate funding that is allocated towards research in chronic lung disease that in no way reflects the heavy societal burden of the disease (The Lancet Respiratory Medicine, 2016). This translates to an overwhelming lack of effective therapies for chronic lung diseases. CPFE represents a chronic lung disease syndrome that has higher mortality and morbidity rates than diseases such as COPD or IPF. As for other chronic lung diseases, limited therapeutic options exist outside lung transplantation (Jankowich and Rounds, 2010, 2012; Lin and Jiang, 2015). Thus, there is a need to identify novel treatments that can attenuate features of CPFE and improve the morbidity and mortality rates. In this study, we have characterized an experimental model of CPFE using male 6 month old *Ada^−/−^* mice and demonstrated that they present with features of CPFE, namely fibrotic deposition, airspace enlargement and PH. We have also generated preliminary results that show that 4MU, also known as hymecromone, a US Food and Drug Administration (FDA)-approved drug, is capable of attenuating fibrotic deposition and PH in a murine experimental model of chronic lung injury that recapitulates features of CPFE. Furthermore, our studies using lung tissues derived from upper or lower lobes from explanted lungs of patients with CPFE or from normal lungs that were discarded for transplantation reveal an upregulated adenosinergic response and evidence of increased hyaluronic acid deposition. Evaluation of the potential mechanisms leading to increased hyaluronan in our experimental model point at ADORA2B-mediated HAS3 expression in macrophages, which is supported by histological observation of dual F4/80- and HAS3-positive cells.

There is a lack of experimental models of lung disease that can accurately recapitulate the pathophysiology of chronic lung diseases (Vlahos and Bozinovski, 2015). This is particularly the case for CPFE, for which limited animal models that recapitulate its cardinal features (fibrotic deposition, airspace enlargement and PH) have been described. Features of CPFE have been modeled by treating mice with both the fibrotic agent bleomycin and elastase to induce airspace enlargement (Cattani-Cavalieri et al., 2017; Trajano et al., 2016). Experiments have also been performed treating cigarette-smoke exposed mice with bleomycin or herpesvirus, leading to airspace enlargement and fibrotic matrix deposition (Zhang et al., 2017). In addition, exposure to the halogen gas bromine has also been shown to induce combined airway fibrosis and emphysema (Aggarwal et al., 2018). Although these models are able to recapitulate features of CPFE, they rely on chemical-induced lung injury, which does not accurately represent the etiology of CPFE (Jankowich and Rounds, 2012). Furthermore, these models have not characterized the presence of PH, an important feature of CPFE (Caminati et al., 2013; Cottin, 2013; Cottin et al., 2010; Seeger et al., 2013).

The *Ada^−/−^* mouse model of lung injury was first reported by Blackburn et al. (1998, 2000) and has been demonstrated to recapitulate important features of chronic lung injury such as fibrotic deposition, airspace enlargement and PH (Karmouty-Quintana et al., 2013a; Schneider et al., 2010; Sun et al., 2006). In these studies both male and female littermate *Ada^−/−^* and *Ada^+^* mice were used and PEG-ADA therapy was maintained typically up to postnatal day (PD) 21 whereupon it was discontinued, leading to a rapid increase in extracellular adenosine levels resulting in lung injury observed typically 15 to 20 days after cessation of PEG-ADA therapy (Karmouty-Quintana et al., 2013a; Schneider et al., 2010; Sun et al., 2006). To adequately model CPFE, a disease that primarily affects males (Cottin et al., 2005; Jankowich and Rounds, 2010, 2012), we used male 6 month old *Ada^−/−^* mice that were subjected to a progressive reduction in PEG-ADA therapy, leading to a gradual increase in extracellular adenosine. Using this approach, we aimed to mimic the repeated subclinical injury that is associated with chronic lung diseases. Mice presented with extensive remodeling of the lung parenchyma including airspace enlargement, fibrotic deposition and vascular remodeling, and PH consistent with previous publications (Karmouty-Quintana et al., 2013a; Schneider et al., 2010; Sun et al., 2006). However, the extent of fibrotic injury and features of PH, such as RVSP, appeared to be enhanced in our model.

An important therapeutic finding from our study was that 4MU was capable of inhibiting PH in *Ada^−/−^* mice, consistent with our previous studies using bleomycin-exposed mice that presented with fibrotic deposition and PH (Collum et al., 2017). However, contrary to the study of Collum et al. (2017), our study demonstrates that 4MU is capable of attenuating the fibrotic deposition in *Ada^−/−^* mice. This discrepancy could be explained by the different treatment regimen of 4MU. In the present study, 4MU was administered for 4 weeks whereas in our previous study it was only given for 2 weeks (Collum et al., 2017). The longer treatment regimen may explain the effect of 4MU at inhibiting both PH and fibrotic deposition; this effect is supported by a greater reduction of hyaluronan levels, observed by enzyme-linked immunosorbent assay (ELISA) in BALF, in our present study using *Ada^−/−^* mice compared to previous studies in the bleomycin model of lung fibrosis (Collum et al., 2017). These are important pre-clinical results that demonstrate an FDA-approved drug has the capacity of attenuating cardinal features of CPFE, a disease that currently lacks any effective therapy outside lung transplantation (Jankowich and Rounds, 2012; Lin and Jiang, 2015).

Consistent with previous publications from our group (Karmouty-Quintana et al., 2013a, 2012), we demonstrate that both elevated ADORA2B expression and hyaluronan accumulation are present in our experimental model of lung injury. A novel finding of our study was that HAS3 was the main HAS that was upregulated in our experimental model, and not HAS1 or HAS2, as had previously been reported in models of lung injury (Collum et al., 2017; Karmouty-Quintana et al., 2013a, 2012). Our results are consistent with studies on cigarette-exposed mice, in which increased lung hyaluronan and HAS3 expression was observed (Bracke et al., 2010). Interestingly, elevated HAS3 signals were primarily observed in macrophages in *Ada^−/−^* mice. In addition, increased HAS3 expression in macrophages was ADORA2B-dependent, consistent with previous publications using mice that lacked ADORA2B-expression in the monocyte lineage (Karmouty-Quintana et al., 2015) and from studies demonstrating an ADORA2B-dependent HAS expression in pulmonary artery smooth muscle cells (Collum et al., 2017; Karmouty-Quintana et al., 2012). Treatment with 4MU was able to attenuate HAS3 expression levels, which is consistent with reduced hyaluronan accumulation and macrophages. These results point at macrophages as an important contributor to the development of lung fibrosis and PH, which is consistent with previous studies (Chen et al., 2016; Karmouty-Quintana et al., 2015; Pedroza et al., 2011; Philip et al., 2017) in which alternatively activated macrophages were shown to promote the development of features of chronic lung injury. It is important to mention that increased HAS3 signals were also observed in epithelial and vascular cells in *Ada^−/−^* mice, which could also contribute to increased hyaluronan accumulation. Increased vascular or perivascular expression of HAS3 and subsequent hyaluronan deposition could also contribute directly to vascular remodeling and PH by promoting vascular stiffness and proliferation, as has been previously demonstrated by our group (Collum et al., 2017). Pulmonary artery smooth muscle cell expression of HAS has been shown to be modulated by activation of ADORA2B (Karmouty-Quintana et al., 2012; Mertens et al., 2018). This is in line with our studies showing ADORA2B-mediated macrophage HAS3 expression.

A novel finding of our study was that HAS3-expessing macrophages appeared to contribute to chronic lung injury. This is significant, as typically HAS3 is associated with the generation of low-molecular-weight (LMW) hyaluronan fragments (Itano et al., 1999). These LMW hyaluronan fragments are associated with pathogenic effects (Bracke et al., 2010; Lennon and Singleton, 2011). Importantly, results from our experimental model were consistent with the presence of HAS3-expressing CD68 cells in CPFE that was associated with massive hyaluronan deposition surrounding fibrotic areas and remodeled vessels from CPFE explanted lungs. These results are highly significant as little is known about the pathogenic mediators involved in the pathophysiology of CPFE.

It is important to mention that adenosine is a danger-associated molecular pattern that is generated following cell injury and the subsequent extracellular release of ATP that is converted to adenosine by CD39 and CD73, with the latter being the rate-limiting enzyme (Colgan et al., 2006). Adenosine can then activate any of its four receptors: ADORA1, ADORA2A, ADORA2B and ADORA3 (Fredholm et al., 2001). Adenosine is known to have many protective effects acutely; however, chronically elevated levels of adenosine and subsequent activation of its low-affinity receptor, ADORA2B, has been extensively associated with chronic lung injury (Karmouty-Quintana et al., 2013b). An important phenomenon that promotes adenosine accumulation is purinergic remodeling, which is characterized by increased expression of CD73, and reduced ADA and ENTs that collectively promote extracellular adenosine accumulation and subsequent ADORA2B activation (Zhou et al., 2009). This phenomenon has been shown to be present in chronic lung diseases such as COPD and IPF (Zhou et al., 2010), and our data from CPFE explanted lung tissue reveal evidence of purinergic remodeling consistent with increased ADORA2B expression. Taken together, these observations advocate the use of an ADORA2B antagonist to treat chronic lung diseases. Indeed, preclinical studies using both *Ada^−/−^* and bleomycin-treated mice have demonstrated that genetic or pharmacological abrogation of ADORA2B signaling is capable of attenuating features of chronic lung injury (Karmouty-Quintana et al., 2013a, 2012; Mustafa et al., 2007; Sun et al., 2006).

Remarkably, treatment with 4MU was not able to attenuate airspace enlargement, RVH or metabolic changes in *Ada^−/−^* mice. RVH is typically a response to increased RVSP; as such, it is conceivable for changes to be observed over time following normalization of blood pressure. Airspace enlargement may be due, however, to other adenosinergic mechanisms that promote alveolar cell destruction. These potential mechanisms may include elevations in deoxy-adenosine (dAdo), a molecule that is also metabolized by ADA and accumulates in *Ada^−/−^* mice (Weng et al., 2013). Increased phosphorylation of dAdo by deoxy-cytosine kinase (DCK) has been shown to contribute to airspace enlargement in *Ada^−/−^* mice and increased DCK signals have been detected in stage 4 COPD (Weng et al., 2013). Taken together, these studies suggest that treatment of 4MU may not alter DCK or dAdo levels to alter airspace enlargement. Similarly, alterations in metabolism have also been reported in *Ada^−/−^* mice (Blackburn et al., 2000) that may not be affected by 4MU, but this compound may be effective at treating lung fibrosis and PH in CPFE.

In conclusion, our study has demonstrated that a gradual reduction of PEG-ADA to male 6 month old *Ada^−/−^* mice results in the development of hallmarks of CPFE: airspace enlargement, fibrotic deposition and PH. In addition, we demonstrate, using a therapeutic approach, that the FDA-approved drug 4MU is capable of reducing the extent of fibrotic injury and the features of PH. Our study also proposes a novel mechanism whereby HAS3-positive macrophages contribute to chronic lung injury in *Ada^−/−^* mice. Importantly, observations in our experimental model of CPFE were recapitulated in lung explants from patients with CPFE, in which increased hyaluronan was detected in remodeled areas of the lung, consistent with HAS3-expressing CD68-positive cells. These results are significant as very little is known about the mechanisms that promote injury in CPFE and, as a result, limited therapies exist for this deadly condition. Our study proposes that inhibition of hyaluronan accumulation is a novel potential therapy for CPFE.

## MATERIALS AND METHODS

### Animals

*Ada^−/−^* were generated as previously described (Blackburn et al., 1998). Briefly, to generate *Ada^−/−^* postnatal mice, an ADA minigene that targeted expression specifically to the trophoblast lineage was introduced onto the *Ada^−/−^* background. This was accomplished by intercrossing mice carrying the trophoblast-specific ADA minigene (Tg) with mice heterozygous for the null Ada allele (m1/+). Subsequent intercrosses yielded litters that contained mice harboring the ADA minigene (Tg) that were also homozygous for the null Ada allele (m1/m1). Given that the regulatory elements used targeted Ada expression only to trophoblasts, once born, and with the loss of the placenta, Tg-m1/m1 mice lacked ADA enzymatic activity. *Ada*^−/−^ mice were genotyped as previously described (Blackburn et al., 2003; Sun et al., 2006). Mice homozygous for the *Ada*-null allele were designated *Ada*^−/−^, whereas control mice, designated *Ada*^+^, were heterozygous for the *Ada*-null allele. *Ada*^−/−^ and *Ada*^+^ mice were congenic on a C57BLk/6J background.

Male 24 week old *Ada^−/−^* mice or littermate *Ada^+^* controls were used for all experiments. All mice were housed in ventilated cages equipped with microisolator lids and kept at an ambient temperature of 22°C and in a 12 h dark/light cycle. All mice were ear-tagged and researchers were blinded to the treatment group. Animal care was in accordance with institutional and National Institutes of Health guidelines. All studies were reviewed and approved by the University of Texas Health Science Center at Houston Animal Welfare Committee.

Following consultation with a statistician, our experimental *n* number was set as 5, based on a power analysis (*F*-tests one-way ANOVA) with the following criteria: alpha error, 0.05; power, 0.95; number of groups, 4; f, 1.189. The power and f values were calculated post-hoc using previous data generated by our lab, including pulseox values, RVSP, Fulton Indices and gene expression data for fibrotic markers. G*Power 3.1.9.2 (Universität Düsseldorf, Germany) was used for all the analysis.

### Experimental design

*Ada*^−/−^ mice were identified at birth and maintained on ADA enzyme therapy from PD2 until PD25 as follows: PD5, 1.25 UI intramuscular (IM); PD9-PD17, 2.5 UI IM; PD21, 2.5 UI intraperitoneal (IP); PD25, 5 UI IP. Starting on PD25, mice received weekly doses of PEG-ADA (5UI IP) until PD168 (week 24). Mice were randomized to group treatment using a random number generator using www.graphpad.com/quickcalcs. Starting on week 24, PEG-ADA was gradually reduced over the course of 9 weeks to 0.125 UI IP as follows: week 1, 5UI IP; week 2, 2.5UI IP; weeks 3 and 4, 1.25UI IP; weeks 5 and 6, 0.625UI IP; weeks 7 and 8, 0.312 UI IP; week 9, 0.125UI IP. On week 34, mice were provided with medicated chow containing 4MU (Sigma-Aldrich) at 20 mg kg^−1^ body weight (BW) per day or normal chow (vehicle; Teklad) for 4 weeks. During the course of these 4 weeks, mice were kept on 0.0125 UI IP of PEG-ADA. The dose was chosen based on previous experiments from our group that demonstrated a beneficial effect at reducing PH in bleomycin-treated mice (Collum et al., 2017). On PD266 (week 38), physiological readouts were performed and the animals were sacrificed for the collection of tissues and fluids for analysis (details below).

### Arterial oxygen saturation

Physiological assessment measuring arterial oxygen saturation was conducted on conscious mice using the pulse MouseOx software analysis (STARR Life Sciences). The hair around the neck was removed from mice in order to use the collar clip light sensor. The MouseOx provides real-time percent oxygen saturation of functional arterial hemoglobin using pulse oximetry measurements of light absorption from the red and infrared LEDs.

### Hemodynamic measurements

RVSP, heart rate and RV hypertrophy measurement was performed as previously described (Karmouty-Quintana et al., 2012). Briefly, mice were given 0.75 mg g^−1^ of 5% Avertin (a mixture of tert-amyl alcohol and 2-2-2 tribromoethanol, Sigma-Aldrich) to induce a surgical plane of anesthesia. Mice were placed on a heated surgical workstation (Molecular Imaging Products) and secured with surgical tape. Mice were then tracheotomised with a 19G blunt needle (Brico Medical Supplies), attached to a small animal ventilator (MiniVent, Hugo-Sachs Elektronik) and ventilated at 250 µl at 200 strokes per minute. The surgical site was viewed using a surgical microscope (SMZ-2B, Nikon). An incision of ∼1 cm in length was made just below the xiphoid process. An alm retractor (ALM-112, Braintree Scientific) was used to expose the abdominal cavity to visualize the diaphragm and the liver. An incision was then made on the diaphragm to expose the heart, and the pericardium was removed. The right ventricle (RV) was then identified and a puncture was made with a 28G needle. A 1 French pressure catheter (SPR-1000, Millar) was then inserted through the puncture. Using the same procedure, we recorded left ventricular systolic pressure (LVSP) immediately after RVSP measurements. The heart rate results were continuously recorded using a Powerlab 8-SP A/D (AD Instruments) converter acquired at 1000 Hz. All RVSP results were recorded to a PC using Chart8 software. After completion of the measurements, blood was collected, and the lungs were excised and formalin fixed paraffin embedded (FFPE). The heart was excised and the atria were removed. The RV was then surgically removed and the dry weights of the RV were used to determine the extent of RV-hypertrophy [RV/left ventricle (LV)+septum]. A heart rate below 250 beats per minute (BPM) during the determination of the RVSP or LVSP was considered a violation of a predetermined criteria.

### Human tissue collection

The use of human material for this study was reviewed by the University of Texas Health Science Center at Houston Committee for the Protection of Human Subjects (Institutional Review Board #HSC-MS-08-0354). Informed consent was obtained to collect all tissues in these studies. The demographic details of the population are summarized in Table S1. Explanted lung tissue from patients with a diagnosis of CPFE or from discarded donor lungs for transplantation were processed on site within 20 min. A 1 cm transverse center cut was obtained from each lobe. This section was divided into portions that were FFPE or flash frozen as previously described (Garcia-Morales et al., 2016).

### RT-PCR and protein expression

Total RNA was isolated from frozen lung tissue using Trizol reagent (Life Technologies) for human samples. Recover All (Thermo Fisher Scientific) was used to isolate RNA from FFPE mouse samples. RNA samples were then DNase treated (ArticZymes) and subjected to quantitative RT-PCR. See Table S2 for primer sequences.

Results were normalized to 18srRNA. Here, two technical replicates were performed per sample. Before RT-PCR experiments, RNA quality was evaluated using the 260 nm/280 nm absorbance values. Samples with 260/280 absorbance ratios below 1.90 or above 2.10 were considered to have poor RNA quality and thus violated a predetermined criteria.

### Liquid chromatography tandem mass spectrometry (LC-MS/MS) analysis of 4MUG concentrations in mouse plasma

For sample preparation, 4-methylumbelliferone-13C4 (Toronto Research Chemicals) and 7-hydroxy coumarin β-D-glucuronide (as sodium salt) (Toronto Research Chemicals) were used as the internal standard (IS) for 4MU and 4MUG, respectively. The neat stock solutions of a mixture of 4MU and 4MUG were diluted in 50% methanol to prepare the spiking solutions ranging from 1 ng/ml to 5000 ng/ml. For calibration standards, 25 µl of blank plasma was mixed with 25 µl of the spiking solutions. For unknown samples, 25 µl of test plasma was mixed with 25 µl of 50% methanol to make up the volume; 25 µl of a mixture of the two IS (1000 ng/ml in 50% methanol) was also added. After vortexing all standards and samples, 150 µl of methanol/acetonitrile 20:80 (v/v) was added to the mixture, which was further vortexed vigorously for 1 min followed by centrifugation at 800 ***g*** for 10 min. Following this, 100 µl of the supernatant was diluted with 200 µl of Milli Q water.

The LC-MS/MS system consisted of an AB SCIEX QTRAP 4000 mass spectrometer coupled to a Shimadzu UFLC system. Mobile phase A is high performance liquid chromatography (HPLC)-grade water. Mobile phase B is HPLC-grade acetonitrile. Liquid chromatography separation was carried out using a Phenomenex Luna PFP(2) column (3 µm, 150×2 mm) with isocratic elution using 45% mobile phase B and a flow rate of 0.4 ml/min at room temperature. The analysis time was 2.5 min. We injected 5 µl of the extracted sample. The mass spectrometer was operated in the negative mode with multiple-reaction monitoring (MRM). The following MRM transitions were used: 4MU (m/z 174.7→132.9), IS for 4MU (m/z 178.7→134.9), 4MUG (m/z 350.8→174.9) and IS for 4MUG (m/z 336.9→160.9). Data acquisition and analysis were performed using the Analyst 1.6.1 software (AB SCIEX).

### Western blots and ELISA

For western blots, protein from lung tissue lysates or pulmonary artery smooth muscle cells (PASMC) was extracted using RIPA buffer (Thermo Fisher Scientific) containing 1 mM of protease and phosphate inhibitor (Sigma-Aldrich). We then loaded 30 μg of protein per sample onto 4-12% Mini-Protean TGX gels (Bio-Rad) for electrophoresis, which was then transferred on polyvinylidene difluoride (PVDF) membranes (0.45 μm, GE Healthcare). Membranes were blocked in 5% milk (Bio-Rad) for 1 h at room temperature and then incubated with the appropriate primary antibody overnight. Secondary antibodies and an ECL Prime Western Blotting kit from GE Healthcare were applied to generate chemiluminescent signals. The following antibodies were used: ADORA2B (rabbit polyclonal, Novus Biologicals, NBP2-41312; 1:2000); HAS3 (rabbit polyclonal, Proteintech, 15609-1-AP; 1:1000); GAPDH (mouse monoclonal, Invitrogen, AM4300; 1:500).

BALF was extracted by flushing the lungs with 0.5 ml of ice-cold PBS twice and collecting the fluid using a 1 ml syringe. The presence of blood in BALF was considered a violation of pre-established criteria. BALF hyaluronan levels were measured by ELISA using a kit from Echelon Biosciences and two technical replicates were performed per sample.

### Histology and IHC

Formalin-fixed-paraffin embedded (FFPE) lung sections were prepared and stained as previously described (Karmouty-Quintana et al., 2013a) and developed using Vector Laboratories NovaRED peroxidase, Vector Red and Vector Blue Alkaline Phosphatase substrate kits. The following primary antibodies were used: smooth muscle actin (mouse monoclonal, clone 1a1, Sigma-Aldrich; 1:1000); HAS3 (rabbit polyclonal, Proteintech, 15609-1-AP; 1:200); rat anti-mouse F4/80 (Bio-Rad, MCA497, clone CI:A3-1; 1:200); rabbit anti-CD68 (Abcam, ab125212; 1:100). To detect hyaluronic acid, sections were incubated with 4 µg/ml of biotinylated HA-binding protein (HABP, Calbiochem; 1:125) overnight at 4°C, incubated with the ABC kit and developed with Vector Blue. For immunofluorescence, sections were incubated with αSMA, incubated with Alexa Fluor 488 or Alexa Fluor 555 (1:500, Sigma-Aldrich), counterstained and mounted with DAPI or PI mounting media (Sigma-Aldrich).

To determine fibrotic deposition, lung sections were stained for Masson's Trichrome and analyzed using a modified Ashcroft scale optimized for mouse lung sections (Hubner et al., 2008). Ten images per animal were analyzed by two individuals blinded to group status.

To quantify airspace enlargement, alveolar airspace size was determined in pressure-infused lungs by measuring mean chord lengths on Hematoxylin and Eosin-stained lung sections. Representative images (black and white) were digitized, and a grid consisting of 53 black lines at 10.5 µm intervals was overlaid on each image. This line grid was subtracted from the black and white lung images using Image-Pro Plus image analysis software (version 2.0, MediaCybernetics). The resultant lines were measured (in µm) and averaged to give the mean chord length of the airspace enlargement. The final mean chord lengths represent averages from ten non-overlapping images of each lung specimen. All quantitative studies were performed blinded with regard to animal genotype and treatment. It is important to note that, for the quantification of airspace enlargement, the images taken were from the parenchyma, devoid of large airway, vascular structures or fibrotic deposition. The exclusion of these structures is necessary to quantify airspace enlargement. However, these exclusions were not performed for the determination of Ashcroft scores.

### Morphometric evaluation of vascular αSMA deposition

Muscularized arterioles of the lung parenchyma were observed under 20× magnification and noted as being different from both airways and non-muscularized arterioles. They were then photographed under 40× magnification and the micropictographs were analyzed using Image Pro-Plus software (MediaCybernetics). In short, the overall area of the muscularized portion was measured for each arteriole. To account for size, the largest diameter for each arteriole was also measured. The area of the arteriole was then divided by the largest diameter to give a relative measurement of muscularization. In additional observations, ten micropictographs of the parenchymal area were taken at 20× magnification. The number of muscularized vessels (those positive for αSMA) were counted for each micropictograph and the total number per animal was obtained and averaged within the group.

### Cell culture experiments

The mouse alveolar macrophage cell line MH-S (ATCC) was maintained at 37°C and 5% CO_2_. Cells were tested for contamination before experimentation. MH-S cells were grown in RPMI-1640 medium supplemented with 10% heat-inactivated fetal bovine serum (FBS), 2 mM L-glutamine and penicillin-streptomycin. For experimental use, MH-S were serum-starved overnight in RPMI-1640 supplemented with 2% FBS, 2 mM L-glutamine and penicillin-streptomycin, followed by 24 h exposure to ADORA2B agonist BAY60-6583 (10 µM, Tocris Bioscience) with or without the ADORA2B antagonist GS6201 (100 nM, Tocris Bioscience).

### Statistical analysis

All analyses were blinded to the experimenter. A two-way ANOVA with the Benjamin, Krieger and Yekutieli post hoc test was performed for all experiments with more than two groups. For experiments that consisted of two groups, an unpaired two-tailed Student's *t*-test with a Welch correction was performed. The Grubbs' test was used to detect outliers. GraphPad Prism 7.0 or higher was used to analyze the data. All software used in this study is available commercially.

## Supplementary Material

Supplementary information

## References

[DMM038711C1] Aggarwal S., Ahmad I., Lam A., Carlisle M. A., Li C., Wells J. M., Raju S. V., Athar M., Rowe S. M., Dransfield M. T. et al. (2018). Heme scavenging reduces pulmonary endoplasmic reticulum stress, fibrosis, and emphysema. *JCI Insight* 3, 120694 10.1172/jci.insight.120694 30385726 PMC6238745

[DMM038711C2] Archer S. L., Weir E. K. and Wilkins M. R. (2010). Basic science of pulmonary arterial hypertension for clinicians: new concepts and experimental therapies. *Circulation* 121, 2045-2066. 10.1161/CIRCULATIONAHA.108.847707 20458021 PMC2869481

[DMM038711C3] Auerbach O., Garfinkel L. and Hammond E. C. (1974). Relation of smoking and age to findings in lung parenchyma: a microscopic study. *Chest* 65, 29-35. 10.1378/chest.65.1.29 4809331

[DMM038711C4] Blackburn M. R., Datta S. K. and Kellems R. E. (1998). Adenosine deaminase-deficient mice generated using a two-stage genetic engineering strategy exhibit a combined immunodeficiency. *J. Biol. Chem.* 273, 5093-5100. 10.1074/jbc.273.9.5093 9478961

[DMM038711C5] Blackburn M. R., Volmer J. B., Thrasher J. L., Zhong H., Crosby J. R., Lee J. J. and Kellems R. E. (2000). Metabolic consequences of adenosine deaminase deficiency in mice are associated with defects in alveogenesis, pulmonary inflammation, and airway obstruction. *J. Exp. Med.* 192, 159-170. 10.1084/jem.192.2.159 10899903 PMC2193256

[DMM038711C6] Blackburn M. R., Lee C. G., Young H. W., Zhu Z., Chunn J. L., Kang M. J., Banerjee S. K. and Elias J. A. (2003). Adenosine mediates IL-13-induced inflammation and remodeling in the lung and interacts in an IL-13-adenosine amplification pathway. *J. Clin. Invest.* 112, 332-344. 10.1172/JCI200316815 12897202 PMC166289

[DMM038711C7] Bracke K. R., Dentener M. A., Papakonstantinou E., Vernooy J. H. J., Demoor T., Pauwels N. S., Cleutjens J., Suylen R. J. v., Joos G. F., Brusselle G. G. et al. (2010). Enhanced deposition of low-molecular-weight hyaluronan in lungs of cigarette smoke–exposed mice. *Am. J. Respir. Cell Mol. Biol.* 42, 753-761. 10.1165/rcmb.2008-0424OC 19675307

[DMM038711C8] Caminati A., Cassandro R. and Harari S. (2013). Pulmonary hypertension in chronic interstitial lung diseases. *Eur. Respir Rev.* 22, 292-301. 10.1183/09059180.00002713 23997057 PMC9487353

[DMM038711C9] Cattani-Cavalieri I., Reis A. G., Kennedy-Feitosa E., Pinho-Ribeiro V., Lanzetti M., Gitirana L. B., Romana-Souza B., Porto L. C. and Valença S. S. (2017). Pulmonary emphysema cross-linking with pulmonary fibrosis and vice versa: a non-usual experimental intervention with Elastase and Bleomycin. *Inflammation* 40, 1487-1496. 10.1007/s10753-017-0590-9 28534139

[DMM038711C11] Chen N. Y., Collum S. D., Luo F., Weng T., Le T. T., Hernandez A. M., Philip K., Molina J. G., Garcia-Morales L. J., Cao Y. et al. (2016). Macrophage bone morphogenic protein receptor 2 depletion in idiopathic pulmonary fibrosis and Group III pulmonary hypertension. *Am. J. Physiol. Lung Cell. Mol. Physiol.* 311, L238-L254. 10.1152/ajplung.00142.2016 27317687 PMC6425517

[DMM038711C12] Chunn J. L., Molina J. G., Mi T., Xia Y., Kellems R. E. and Blackburn M. R. (2005). Adenosine-dependent pulmonary fibrosis in adenosine deaminase-deficient mice. *J. Immunol.* 175, 1937-1946. 10.4049/jimmunol.175.3.1937 16034138

[DMM038711C13] Colgan S. P., Eltzschig H. K., Eckle T. and Thompson L. F. (2006). Physiological roles for ecto-5’-nucleotidase (CD73). *Purinergic Signal* 2, 351-360. 10.1007/s11302-005-5302-5 18404475 PMC2254482

[DMM038711C14] Collum S. D., Chen N.-Y., Hernandez A. M., Hanmandlu A., Sweeney H., Mertens T. C. J., Weng T., Luo F., Molina J. G., Davies J. et al. (2017). Inhibition of hyaluronan synthesis attenuates pulmonary hypertension associated with lung fibrosis. *Br. J. Pharmacol.* 174, 3284-3301. 10.1111/bph.13947 28688167 PMC5595757

[DMM038711C15] Cottin V. (2013). The impact of emphysema in pulmonary fibrosis. *Eur. Respir Rev.* 22, 153-157. 10.1183/09059180.00000813 23728869 PMC9487383

[DMM038711C16] Cottin V., Nunes H., Brillet P. Y., Delaval P., Devouassoux G., Tillie-Leblond I., Israel-Biet D., Court-Fortune I., Valeyre D. and Cordier J. F. (2005). Combined pulmonary fibrosis and emphysema: a distinct underrecognised entity. *Eur. Respir. J.* 26, 586-593. 10.1183/09031936.05.00021005 16204587

[DMM038711C17] Cottin V., Le Pavec J., Prevot G., Mal H., Humbert M., Simonneau G. and Cordier J. F. (2010). Pulmonary hypertension in patients with combined pulmonary fibrosis and emphysema syndrome. *Eur. Respir. J.* 35, 105-111. 10.1183/09031936.00038709 19643948

[DMM038711C18] Fredholm B. B., AP I. J., Jacobson K. A., Klotz K. N. and Linden J. (2001). International Union of Pharmacology. XXV. Nomenclature and classification of adenosine receptors. *Pharmacol. Rev.* 53, 527-552. 11734617 PMC9389454

[DMM038711C19] Garcia-Morales L. J., Chen N.-Y., Weng T., Luo F., Davies J., Philip K., Volcik K. A., Melicoff E., Amione-Guerra J., Bunge R. R. et al. (2016). Altered hypoxic-adenosine axis and metabolism in group iii pulmonary hypertension. *Am. J. Respir. Cell Mol. Biol.* 54, 574-583. 10.1165/rcmb.2015-0145OC 26414702 PMC4821053

[DMM038711C20] Hogg J. C. and Timens W. (2009). The pathology of chronic obstructive pulmonary disease. *Annu. Rev. Pathol.* 4, 435-459. 10.1146/annurev.pathol.4.110807.092145 18954287

[DMM038711C21] Hubner R. H., Gitter W., El Mokhtari N. E., Mathiak M., Both M., Bolte H., Freitag-Wolf S. and Bewig B. (2008). Standardized quantification of pulmonary fibrosis in histological samples. *BioTechniques* 44, 507-511. 10.2144/000112729 18476815

[DMM038711C22] Itano N., Sawai T., Yoshida M., Lenas P., Yamada Y., Imagawa M., Shinomura T., Hamaguchi M., Yoshida Y., Ohnuki Y. et al. (1999). Three isoforms of mammalian hyaluronan synthases have distinct enzymatic properties. *J. Biol. Chem.* 274, 25085-25092. 10.1074/jbc.274.35.25085 10455188

[DMM038711C23] Jankowich M. D. and Rounds S. (2010). Combined pulmonary fibrosis and emphysema alters physiology but has similar mortality to pulmonary fibrosis without emphysema. *Lung* 188, 365-373. 10.1007/s00408-010-9251-6 20614219 PMC2939964

[DMM038711C24] Jankowich M. D. and Rounds S. I. S. (2012). Combined pulmonary fibrosis and emphysema syndrome: a review. *Chest* 141, 222-231. 10.1378/chest.11-1062 22215830 PMC3251269

[DMM038711C25] Karmouty-Quintana H., Philip K., Acero L. F., Chen N.-Y., Weng T., Molina J. G., Luo F., Davies J., Le N.-B., Bunge I. et al. (2015). Deletion of ADORA2B from myeloid cells dampens lung fibrosis and pulmonary hypertension. *FASEB J.* 29, 50-60. 10.1096/fj.14-260182 25318478 PMC4763976

[DMM038711C26] Karmouty-Quintana H., Zhong H., Acero L., Weng T., Melicoff E., West J. D., Hemnes A., Grenz A., Eltzschig H. K., Blackwell T. S. et al. (2012). The A2B adenosine receptor modulates pulmonary hypertension associated with interstitial lung disease. *FASEB J.* 26, 2546-2557. 10.1096/fj.11-200907 22415303 PMC3650483

[DMM038711C27] Karmouty-Quintana H., Weng T., Garcia-Morales L. J., Chen N.-Y., Pedroza M., Zhong H., Molina J. G., Bunge R., Bruckner B. A., Xia Y. et al. (2013a). Adenosine A2B receptor and hyaluronan modulate pulmonary hypertension associated with chronic obstructive pulmonary disease. *Am. J. Respir. Cell Mol. Biol.* 49, 1038-1047. 10.1165/rcmb.2013-0089OC 23855769 PMC5459551

[DMM038711C28] Karmouty-Quintana H., Xia Y. and Blackburn M. R. (2013b). Adenosine signaling during acute and chronic disease states. *J. Mol. Med. (Berl.)* 91, 173-181. 10.1007/s00109-013-0997-1 23340998 PMC3606047

[DMM038711C29] Le T.-T. T., Karmouty-Quintana H., Melicoff E., Le T.-T. T., Weng T., Chen N.-Y., Pedroza M., Zhou Y., Davies J., Philip K. et al. (2014). Blockade of IL-6 trans signaling attenuates pulmonary fibrosis. *J. Immunol.* 193, 3755-3768. 10.4049/jimmunol.1302470 25172494 PMC4169999

[DMM038711C30] Lennon F. E. and Singleton P. A. (2011). Role of hyaluronan and hyaluronan-binding proteins in lung pathobiology. *Am. J. Physiol. Lung Cell. Mol. Physiol.* 301, L137-L147. 10.1152/ajplung.00071.2010 21571904 PMC3154626

[DMM038711C31] Lin H. and Jiang S. (2015). Combined pulmonary fibrosis and emphysema (CPFE): an entity different from emphysema or pulmonary fibrosis alone. *J. Thorac. Dis.* 7, 767-779. 25973246 10.3978/j.issn.2072-1439.2015.04.17 PMC4419325

[DMM038711C32] Luo F., Le N.-B., Mills T., Chen N.-Y., Karmouty-Quintana H., Molina J. G., Davies J., Philip K., Volcik K. A., Liu H. et al. (2016). Extracellular adenosine levels are associated with the progression and exacerbation of pulmonary fibrosis. *FASEB J.* 30, 874-883. 10.1096/fj.15-274845 26527068 PMC4714555

[DMM038711C33] Lynch J. and Toews G. (1998). Idiopathic pulmonary fibrosis. In *Pulmonary Diseases and Disorders*, Vol. 1 (ed. Fishman A. P., Elias J. A., Fishman J. A., Grippi M. A., Kaiser L. R. and Senior R. M.), pp. 1069-1084. New york: McGraw-Hill.

[DMM038711C34] Mertens T. C. J., Hanmandlu A., Tu L., Phan C., Collum S. D., Chen N. Y., Weng T., Davies J., Liu C., Eltzschig H. K. et al. (2018). Switching-off Adora2b in vascular smooth muscle cells halts the development of pulmonary hypertension. *Front. Physiol.* 9, 555 10.3389/fphys.2018.00555 29910735 PMC5992271

[DMM038711C35] Mustafa S. J., Nadeem A., Fan M., Zhong H., Belardinelli L. and Zeng D. (2007). Effect of a specific and selective A(2B) adenosine receptor antagonist on adenosine agonist AMP and allergen-induced airway responsiveness and cellular influx in a mouse model of asthma. *J. Pharmacol. Exp. Ther.* 320, 1246-1251. 10.1124/jpet.106.112250 17159162

[DMM038711C36] Panos R. J., Mortenson R. L., Niccoli S. A. and King T. E. Jr. (1990). Clinical deterioration in patients with idiopathic pulmonary fibrosis: causes and assessment. *Am. J. Med.* 88, 396-404. 10.1016/0002-9343(90)90495-Y 2183601

[DMM038711C37] Pauwels R. A., Buist A. S., Calverley P. M., Jenkins C. R. and Hurd S. S. (2001). Global strategy for the diagnosis, management, and prevention of chronic obstructive pulmonary disease. NHLBI/WHO global initiative for chronic obstructive lung disease (GOLD) workshop summary. *Am. J. Respir. Crit. Care. Med.* 163, 1256-1276. 10.1164/ajrccm.163.5.2101039 11316667

[DMM038711C38] Pedroza M., Schneider D. J., Karmouty-Quintana H., Coote J., Shaw S., Corrigan R., Molina J. G., Alcorn J. L., Galas D., Gelinas R. et al. (2011). Interleukin-6 contributes to inflammation and remodeling in a model of adenosine mediated lung injury. *PLoS ONE* 6, e22667 10.1371/journal.pone.0022667 21799929 PMC3143181

[DMM038711C39] Philip K., Mills T. W., Davies J., Chen N.-Y., Karmouty-Quintana H., Luo F., Molina J. G., Amione-Guerra J., Sinha N., Guha A. et al. (2017). HIF1A up-regulates the ADORA2B receptor on alternatively activated macrophages and contributes to pulmonary fibrosis. *FASEB J.* 31, 4745-4758. 10.1096/fj.201700219R 28701304 PMC5636704

[DMM038711C40] Schneider D. J., Lindsay J. C., Zhou Y., Molina J. G. and Blackburn M. R. (2010). Adenosine and osteopontin contribute to the development of chronic obstructive pulmonary disease. *FASEB J.* 24, 70-80. 10.1096/fj.09-140772 19720619 PMC2797041

[DMM038711C41] Seeger W., Adir Y., Barberà J. A., Champion H., Coghlan J. G., Cottin V., De Marco T., Galiè N., Ghio S., Gibbs S. et al. (2013). Pulmonary hypertension in chronic lung diseases. *J. Am. Coll. Cardiol.* 62, D109-D116. 10.1016/j.jacc.2013.10.036 24355635

[DMM038711C42] Sime P. J. and O'Reilly K. M. A. (2001). Fibrosis of the lung and other tissues: new concepts in pathogenesis and treatment. *Clin. Immunol.* 99, 308-319. 10.1006/clim.2001.5008 11358425

[DMM038711C43] Sun C.-X., Zhong H., Mohsenin A., Morschl E., Chunn J. L., Molina J. G., Belardinelli L., Zeng D. and Blackburn M. R. (2006). Role of A2B adenosine receptor signaling in adenosine-dependent pulmonary inflammation and injury. *J. Clin. Invest.* 116, 2173-2182. 10.1172/JCI27303 16841096 PMC1501110

[DMM038711C44] Thannickal V. J., Toews G. B., White E. S., Lynch J. P. III and Martinez F. J. (2004). Mechanisms of pulmonary fibrosis. *Annu. Rev. Med.* 55, 395-417. 10.1146/annurev.med.55.091902.103810 14746528

[DMM038711C45] The Lancet Respiratory Medicine (2016). Lung disease left out in the cold. *Lancet Respir Med.* 4, 527 10.1016/S2213-2600(16)30155-2 27396761

[DMM038711C46] Trajano L. A. S. N., Trajano E. T. L., Lanzetti M., Mendonça M. S. A., Guilherme R. F., Figueiredo R. T., Benjamim C. F., Valenca S. S., Costa A. M. A. and Porto L. C. (2016). Elastase modifies bleomycin-induced pulmonary fibrosis in mice. *Acta Histochem.* 118, 203-212. 10.1016/j.acthis.2015.12.010 26852294

[DMM038711C47] Vlahos R. and Bozinovski S. (2015). Preclinical murine models of chronic obstructive pulmonary disease. *Eur. J. Pharmacol.* 759, 265-271. 10.1016/j.ejphar.2015.03.029 25818750

[DMM038711C48] Weng T., Karmouty-Quintana H., Garcia-Morales L. J., Molina J. G., Pedroza M., Bunge R. R., Bruckner B. A., Loebe M., Seethamraju H. and Blackburn M. R. (2013). Hypoxia-induced deoxycytidine kinase expression contributes to apoptosis in chronic lung disease. *FASEB J.* 27, 2013-2026. 10.1096/fj.12-222067 23392349 PMC4046114

[DMM038711C49] Zhang W.-G., Wu S.-S., He L., Yang Q., Feng Y.-K., Chen Y.-T., Zhen G.-H., Xu Y.-J., Zhang Z.-X., Zhao J.-P. et al. (2017). Comparative study of two models of combined pulmonary fibrosis and emphysema in mice. *Acta Histochem.* 119, 244-251. 10.1016/j.acthis.2017.01.007 28233574

[DMM038711C50] Zhou Y., Schneider D. J. and Blackburn M. R. (2009). Adenosine signaling and the regulation of chronic lung disease. *Pharmacol. Ther.* 123, 105-116. 10.1016/j.pharmthera.2009.04.003 19426761 PMC2743314

[DMM038711C51] Zhou Y., Murthy J. N., Zeng D., Belardinelli L. and Blackburn M. R. (2010). Alterations in adenosine metabolism and signaling in patients with chronic obstructive pulmonary disease and idiopathic pulmonary fibrosis. *PLoS ONE* 5, e9224 10.1371/journal.pone.0009224 20169073 PMC2821921

