# Supplementary material for: Adenosine and hyaluronan promote lung fibrosis and pulmonary hypertension in combined pulmonary fibrosis and emphysema
Source: Dis Model Mech. 2019 May 15;12(5):dmm038711. doi: 10.1242/dmm.038711 (PMC6550050; doi:10.1242/dmm.038711)
Supplement: Supplementary information [file dmm-12-038711-s1.pdf]

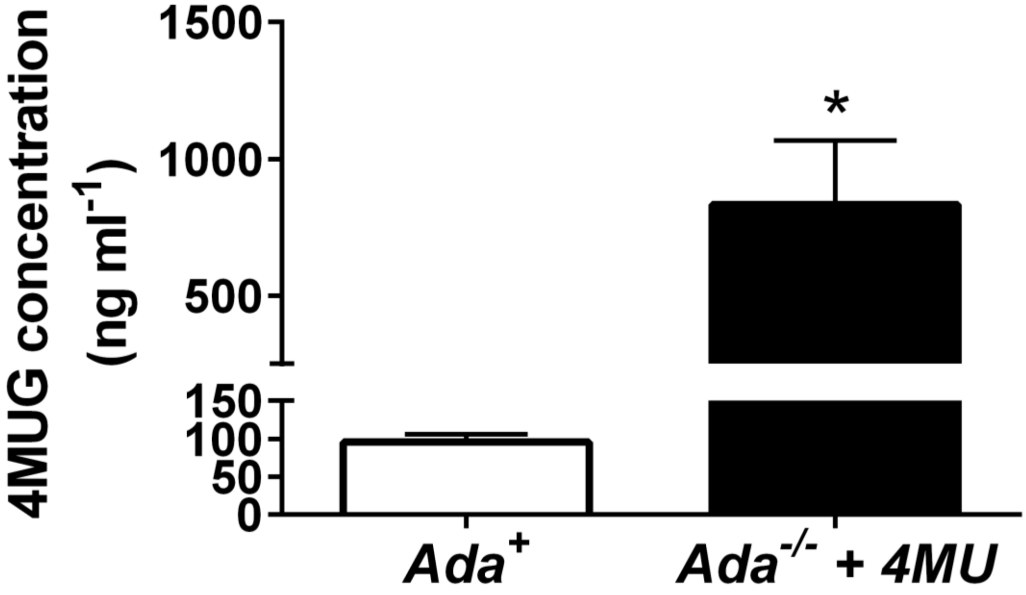

Figure S1. Plasma levels for the 4-methylumbelliferone (4MU) metabolite:

4-Methylumbelliferyl-β-D-glucuronide (4MUG) from *Ada*$^{-/-}$ and *Ada*$^{-/-}$ + 4MU mice. N=5 for all groups. Significance level * P≤0.05 refers to ANOVA comparisons between *Ada*$^{-/-}$ and *Ada*$^{-/-}$ + 4MU treatment groups.

Table S1. Demographic description of the study population

Subject No.	Age	Sex	Ethnicity	BMI	mPAP (mm Hg)	Smoker?	Cause of Death
CPFE 1	51	Male	H	27.3	20	Yes	n/a
CPFE 2	63	Male	C	29.5	19	Yes	n/a
CPFE 3	70	Male	C	26.8	29	No	n/a
CPFE 4	57	Male	C	28.6	28	n/a	n/a
Mean	***60.4***			***28.0***	***24***		
Normal 1	50	Male	C	25.0	n/a	Yes	Head trauma
Normal 2	46	Male	C	34.5	n/a	Yes	Stroke
Normal 3	65	Male	H	26.5	n/a	No	Stroke
Normal 4	56	Male	H	32.7	n/a	n/a	Stroke
Mean	***54.25***			***29.7***			
P Value	0.3407			0.5407			

Definition of abbreviations: Age, age at time of lung transplantation or explantation; BMI, body mass index; mPAP, mean Pulmonary Arterial Pressure measured by right-heart catherization, n/a, not available or applicable. Smoker status refers to P Values were determined through an unpaired t test with Welch's correction.

Table S2. Primers used

Gene	Forward Primer	Reverse Primer
18srRNA	GTAACCCGTTGAACCCCATT	CCATCCAATCGGTAGTAGCG
**Human**		
ADA	GGGCTGCTGAACGTCATTG	AGGCATGTAGTAGTCAAACTTGG
ADORA1A	TGCACTGACTTCTACGGCTG	GGTCCCCGTGACCAAACTT
ADORA2A	CGCTCCGGTACAATGGCTT	TTGTTCCAACCTAGCATGGGA
ADORA2B	TGCACTGACTTCTACGGCTG	GGTCCCCGTGACCAAACTT
ADORA3	TCATTCTACTCTCCTTGGCTCTC	GTGGGCATTGTAGTTGCAGAT
CD39	AGGTGCCTATGGCTGGATTAC	CCAAAGCTCCAAAGGTTTCCT
CD73	CCAGTACCAGGGCACTATCTG	TGGCTCGATCAGTCCTTCCA
COL1A1	GTGCGATGACGTGATCTGTGA	CGGTGGTTTCTTGGTCGGT
COL1A2	GAGCGGTAACAAGGGTGAGC	CTTCCCCATTAGGGCCTCTC
COL2A1	TGGACGCCATGAAGGTTTTCT	TGGGAGCCAGATTGTCATCTC
ENT2	GGGGTACTTTATCACGCCCTG	GGGAATCCCGTTCTCATCAGA
FN1	GGTGGAATAGAGCTCCCAGG	GCAGCCTGCATCTGAGTACA
HAS1	GAGCCTCTTCGCGTACCTG	CCTCCTGGTAGGCGGAGAT
HAS2	TCCAAAGAGTGTGGTTCCAA	GACAGGCTGAGGACGACTTT
HAS3	CGCAGCAACTTCCATGAGG	AGTCGCACACCTGGATGTAGT
IL6	AATTCGGTACATCCTCGACGG	TTGGAAGGTTCAGGTTGTTTTCT
**Mouse**		
Adora2b	GCGTCCCGCTCAGGTATAAAG	CGGAGTCAATCCAATGCCAAAG
Fn	ACTGGATGGGGTGGGAAT	GGAGTGGCACTGTCAACCTC
Has1	GCGAGCACTCACGATCATCTT	GTCCATAGCGATCTGAAGCCA
Has2	ACAGATGAGGCAGGGTCAAG	TGGGGTGGAAAGAGAGAAGT
Has3	GTGGGCACCAGTCTGTTTG	CCACTGAACGCGACCTCTG
Pparg	GGAAGACCACTCGCATTCCTT	GTAATCAGCAACCATTGGGTCA
Sdha	GGAACACTCCAAAAACAGACCT	CCACCACTGGGTATTGAGTAGAA

Disease Models & Mechanisms • Supplementary information
